# Supplementary material for: Evidence for influenza and RSV interaction from 10 years of enhanced surveillance in Nha Trang, Vietnam, a modelling study
Source: PLoS Comput Biol. 2022 Jun 24;18(6):e1010234. doi: 10.1371/journal.pcbi.1010234 (PMC9262224; doi:10.1371/journal.pcbi.1010234)
Supplement: S1 Text — (DOCX) [file pcbi.1010234.s001.docx]

**Supplement for:** Evidence for Influenza and RSV interaction from 10 years of enhanced surveillance in Nha Trang, Vietnam, a modelling study.

**Authors**: Naomi R Waterlow*^1^**, Michiko Toizumi^2^, Edwin van Leeuwen*^1,3^*, Hien-Anh Thi Nguyen4, Lay Myint-Yoshida^2¶^, Rosalind M Eggo*^1^*^¶^, Stefan Flasche*^1^*^¶^.

**Affiliations**:

^1^ Centre for Mathematical Modelling of Infectious Disease, London School of Hygiene and Tropical Medicine, United Kingdom

^2^ Department of Pediatric Infectious Diseases, Institute of Tropical Medicine, Nagasaki University, Nagasaki, Japan

^3^ Statistics, Modelling and Economics Department, UKHSA. London, United Kingdom

^4^ National Institute of Hygiene and Epidemiology, Hanoi, Vietnam

* indicates corresponding author

^¶^ These authors have contributed equally

[**Estimated influenza attack rate**](#_k69saasgej2s) **2**

[**Correlation**](#_tgyfop6it5xp) **2**

[**Model equations**](#_8gd1eaa1wr4g) **3**

[**R0 equations**](#_31b8iib17k68) **4**

[**Susceptibility to RSV**](#_3gt7bd7k5wmz) **5**

[**Susceptibility to Influenza**](#_niwk4p38xwvr) **5**

[**Parallel tempering**](#_ajf9s763zyr3) **6**

[**Attack Rates**](#_9uvzf3i4gqcl) **8**

[**Sensitivity to severity of dual infected cases**](#_48fwsx1ukkq3) **9**

[**Prior Sensitivity**](#_7l9cmgsw79c8) **9**

[**References**](#_hyjmc5y1z7m2) **10**

## Estimated influenza attack rate

Assuming no interaction (in susceptibility to or severity of dual infections), we calculated the required annual influenza infection attack rate in order to achieve the observed number of dual infections (equations 1-3). Using a negative binomial likelihood with Brent optimization we estimated the RSV reporting rate that would correspond to the maximum likelihood of observing the reported weekly number of dual infections. We then used this estimate of the reporting rate to calculate the annual RSV population attack rate required in order to observe this many dual cases. The confidence intervals for the attack rate were calculated using the Hessian matrix from the optimisation.

$I_{Dual} \simeq I_{RSV}* P_{Influenza}$ (1)

$P_{Influenza}\simeq I_{Influenza}* 1/\gamma_{Influenza}/ \upsilon_{Influenza}$ (2)

${AR}_{Influenza}\simeq I_{Influenza}/ \upsilon_{Influenza}$ (3)

With parameters: Incidence of reported cases (I), Prevalence of Infection (P), Duration of Infection$( 1/\gamma$, 3.8 days - see main text Table 1) and estimated reporting rate **(**$\upsilon$).

We estimated that in order to achieve the weekly reported number of dual infections given no interaction, we would require an annual influenza attack rate of 4.4 (3.4 -6.5) in ages 0-1 and 1.3 (0.9 - 3.0) in ages 2-4.
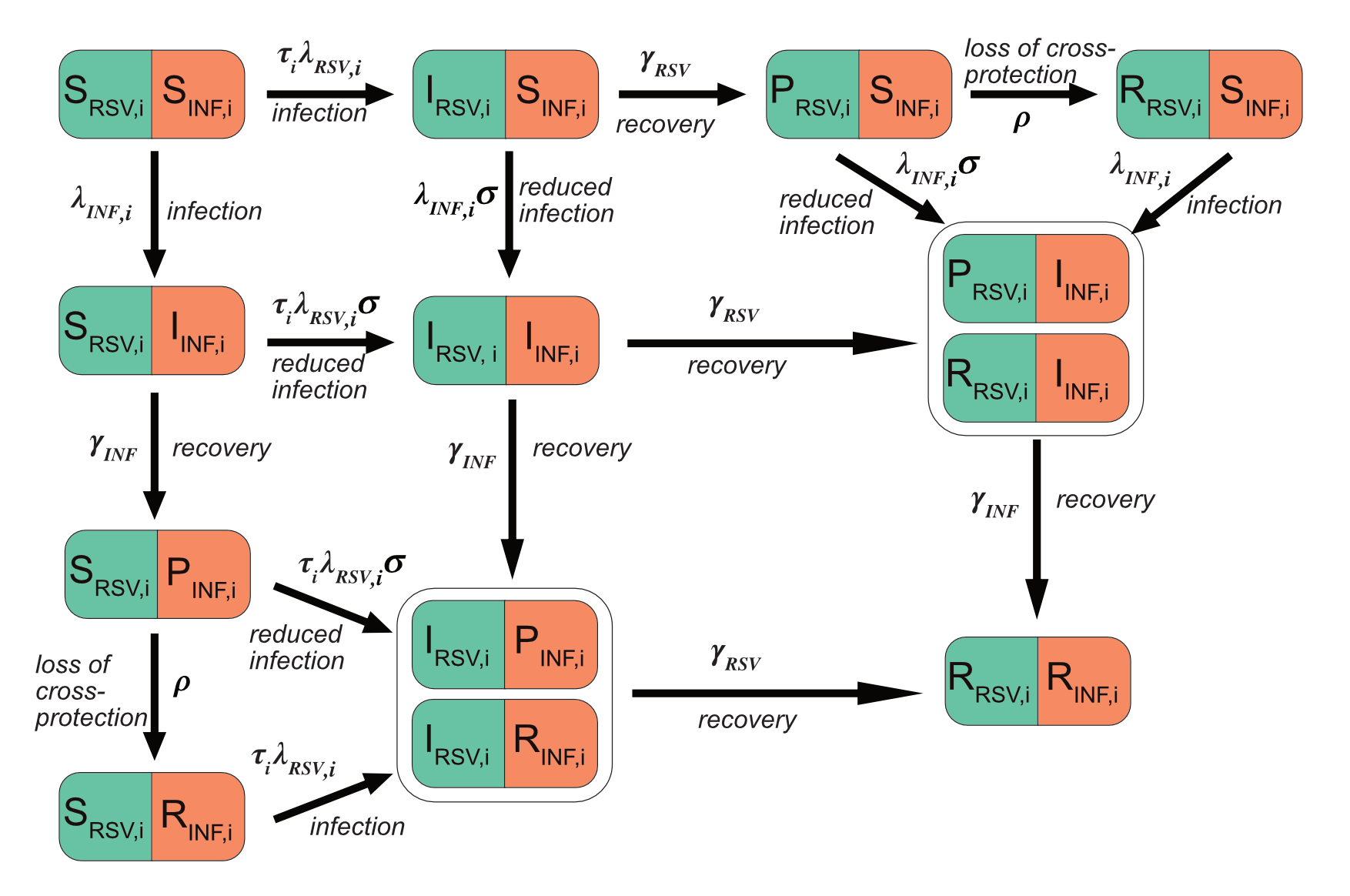


## Correlation

Figure A shows a scatter plot between the weekly Influenza and RSV cases.
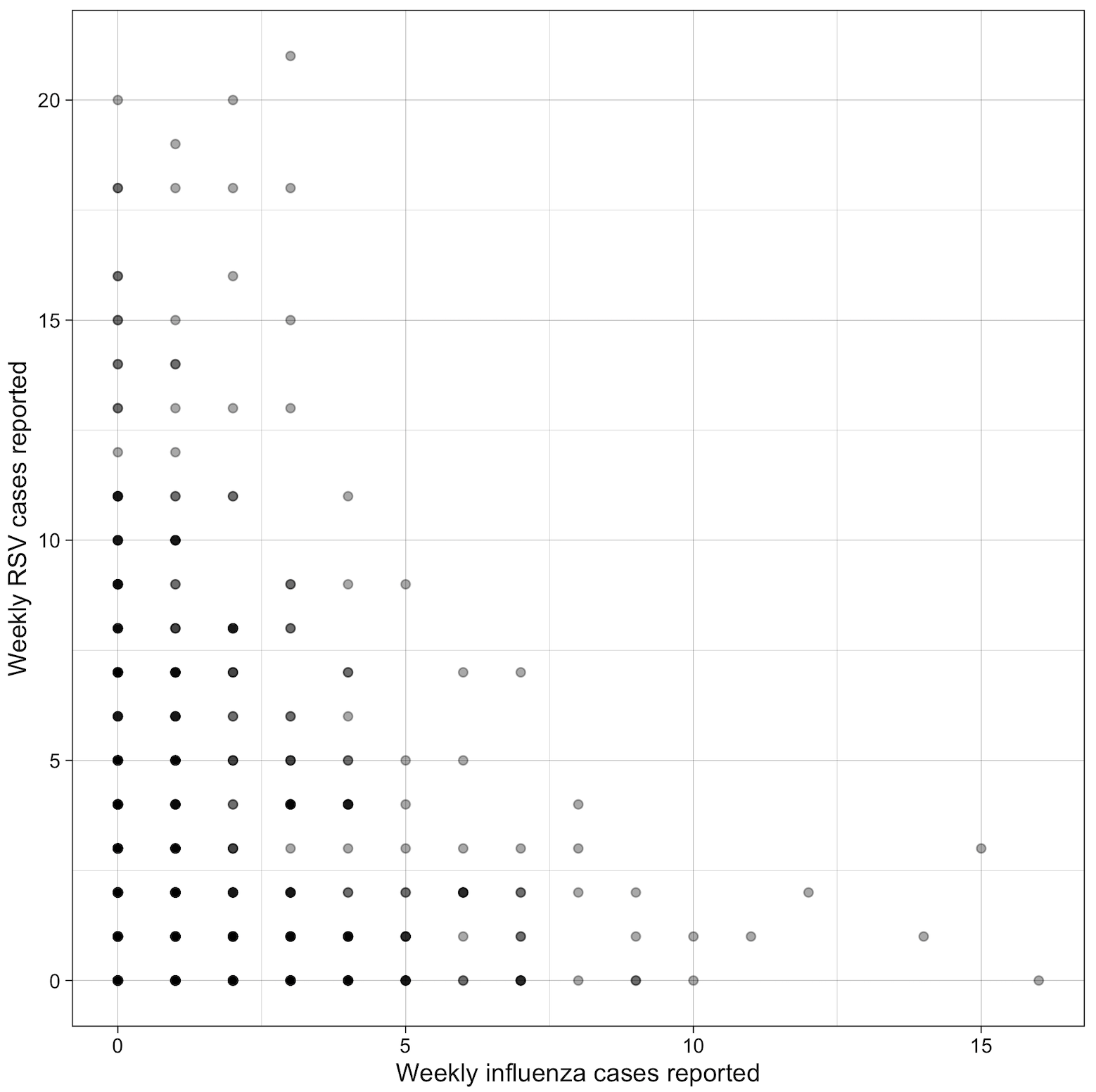


**Figure A: Reported cases**. Scatter plot of weekly influenza and RSV cases reported through the enhanced surveillance study in less than 5 year olds over the whole time period.

## Model equations

Full model equations are shown below. Each compartment includes the state for both RSV and influenza, with the first letter indicating the state for RSV, and the second for influenza. E.g. $SS_{i}$is shorthand for $S_{RSV,i}S_{INF,i}$. Subscripts used are “INF” for influenza and “RSV”.

Susceptibles become infected at force of infections $\lambda_{INF}$ and $\lambda_{RSV}$, and move into the I states. They then remain infectious for $1/\gamma_{INF}$ and $1/\gamma_{RSV}$ days and during the infectious period and $1/\rho$ days thereafter they are cross-protected and thus their propensity for heterologous infection is reduced by factor $\sigma$, the strength of cross-protection. All age groups are equally susceptible to influenza, but there is reduced susceptibility to RSV in older age groups, determined by parameter $\tau_{i}$.

$\lambda_{INF,i} = \sum_{j=1}^{5} \beta_{INF}\alpha_{ij}I_{INF,j}$

$\lambda_{RSV,i} = \sum_{j=1}^{5} \beta_{RSV}\alpha_{ij}I_{RSV,j}$

$$\frac{dSS_{i}}{dt}={- \tau_{i}\lambda}_{{RSV}_{i}}SS_{i} {- \lambda}_{{INF}_{i}}{SS}_{i} - \epsilon_{INF}- \epsilon_{RSV}$$

$$\frac{d{IS}_{i}}{dt}= {\tau_{i}\lambda}_{{RSV}_{i}}{SS}_{i} {- (1-\sigma)\lambda}_{{INF}_{i}}{IS}_{i}-\gamma_{RSV}{IS}_{i} + \epsilon_{RSV}$$

$$\frac{d{PS}_{i}}{dt}= \gamma_{RSV}{IS}_{i}-\rho PS_{i}-(1-\sigma){\lambda_{INF}}_{i}PS_{i}$$

$$\frac{d{RS}_{i}}{dt}= \rho PS_{i}-{\lambda_{INF}}_{i}RS_{i}$$

$$\frac{d{SI}_{i}}{dt}= \lambda_{{INF}_{i}}{SS}_{i}- {{(1-\sigma)\tau}_{i}\lambda}_{{RSV}_{i}}{SI}_{i}-\gamma_{INF}{SI}_{i} + \epsilon_{INF}$$

$$\frac{d{II}_{i}}{dt}= {(1-\sigma)\lambda}_{{INF}_{i}}{IS}_{i}+(1-{\sigma)\tau_{i}\lambda}_{{RSV}_{i}}{SI}_{i}-\gamma_{INF}{II}_{i}- \gamma_{RSV}{II}_{i}$$

$$\frac{d{PI}_{i}}{dt}= (1-\sigma){\lambda_{INF}}_{i}{PS}_{i}-\gamma_{INF}{PI}_{i}+\gamma_{RSV}{II}_{i}+\lambda_{{INF}_{i}}{RS}_{i}$$

$$\frac{d{SP}_{i}}{dt}= \gamma_{INF}{SI}_{i}-\rho{SP}_{i}-(1-\sigma){{\tau_{i}\lambda}_{RSV}}_{i}{SP}_{i}$$

$$\frac{d{IP}_{i}}{dt}= {(1-\sigma)\tau_{i}\lambda}_{RSV}{SP}_{i}-\gamma_{RSV}{IP}_{i}+\gamma_{INF}{II}_{i}+{\tau_{i}\lambda}_{{RSV}_{i}}{SR}_{i}$$

$$\frac{d{SR}_{i}}{dt}= \rho{SP}_{i}-{{\tau_{i}\lambda}_{RSV}}_{i}{SR}_{i}$$

$$\frac{d{RR}_{i}}{dt}= \gamma_{RSV}{IP}_{i}+\gamma_{INF}{PI}_{i}$$

Where:

$\lambda_{i,j}$ - force of infection between age groups I and J

$\beta$- transmission rate

$\alpha_{ij}$- contact rate between group I and j

$\tau_{i}$ - age group susceptibility to RSV

$\sigma$ - level of cross-protection

$\gamma$ - rate of recovery

$\rho$ - rate of loss of cross-protection

$\epsilon$ - infection from external sources

## *R_0_* equations

The R0’s were calculated as the dominant eigenvalue of the matrix

$$-T\Sigma^{-1}$$

Where $T$is the transmission matrix, describing new infections, and$\Sigma$is the transition matrix, describing other changes in state. This method is described in full in Diekmann *et al (2009)*^1^

**
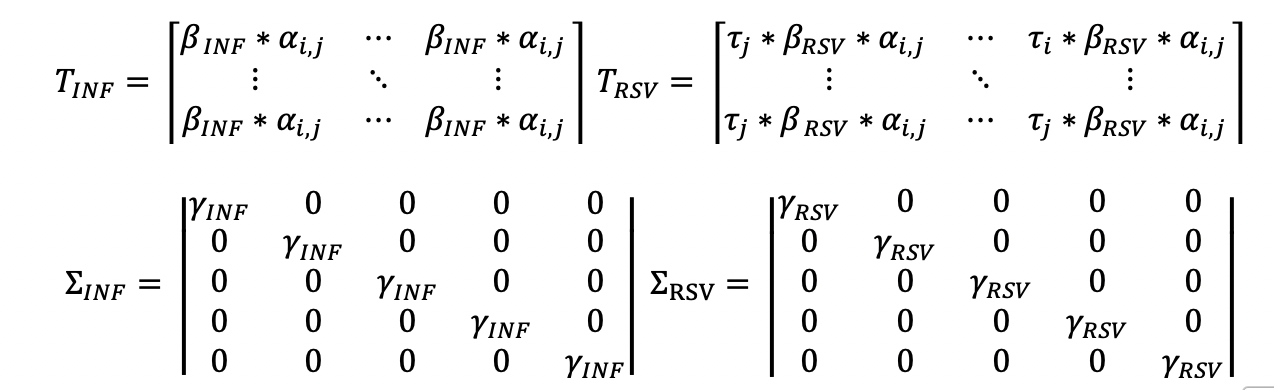
**

The posterior estimates led to *R_0_*s of 1.07 (95%CrI 1.06-1.1) and 1.24 (95%CrI 1.23 - 1.26) for influenza and RSV respectively.

## Susceptibility to RSV

We used a longitudinal study by Hendersen et a. (1979) to determine age-susceptibility to RSV infection. They estimated that at 1st exposure 98.4% of children became infected, at second exposure 74.5% of children became infected and at 3rd exposure 65.4% of children became infected^2^. As most children are infected by 24 months of age, we used the susceptibility estimates for the age groups: ages 0-1 = 100% susceptible, ages 2-4 = 75% susceptible, ages 5 and over = 65% susceptible^3^.

## Susceptibility to Influenza

Influenza susceptibility each season (s) is determined by the parameter $\eta_{s}$using the inverse density of an exponential distribution at each age group (where ages 0-1 is age group zero, up to ages 65+ at age group four). Example susceptibility profiles are shown in Figure B.


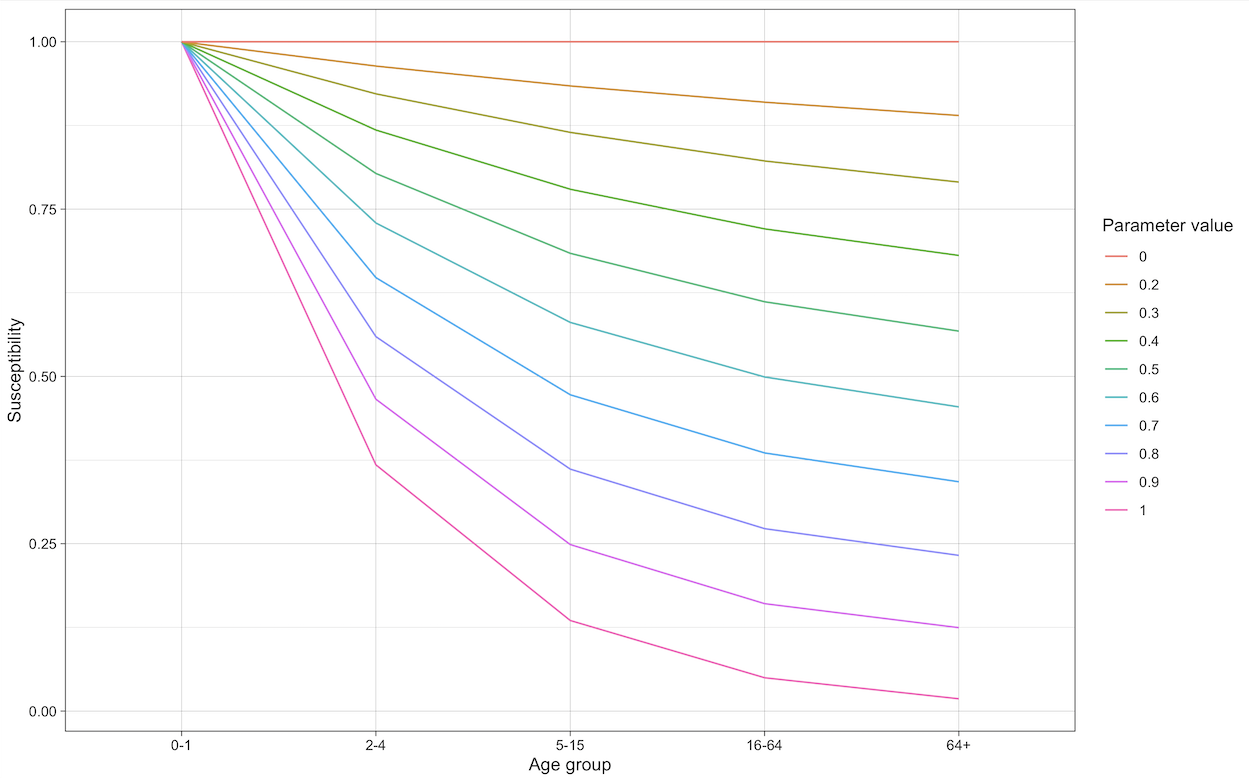


***Figure B: Susceptibility.*** *Susceptibility to influenza by age group for different parameter values.*

## Parallel tempering

Our parallel tempering algorithm was implemented in R and we used Amazon Web Services (AWS) to run it. We proposed swaps with the next temperature chains every 5 iterations. We ran the parallel tempering algorithm using a covariance matrix to propose parameters. We removed 250000 iterations as burn in, followed by 200000 more samples, and assessed convergence using the Geweke statistic in the null chain (Chain with temperature 1). This calculates the difference between the two sample means of the first 10 and last 50% of the chain, divided by its estimated standard error, resulting in a Z score. Note however that due to the large number of parameters (44), the multiple modes and the swapping between chains as a result of the parallel tempering the Geweke statistic is not an ideal measure of convergence in this situation. Despite this, all key parameters (transmission rates, interaction parameters, dual detection rate) had a Z score within the 95% confidence interval and overall over 80% of parameters fell within a 99% confidence interval. Figure C shows one of the traces for a sample of parameters, thinned to 1 in 10. Figure D shows the final posterior densities of each parameter, and Figure E describes the distribution. We also ran a second set of chains starting at different parameter values, which converged on the same parameter spaces.


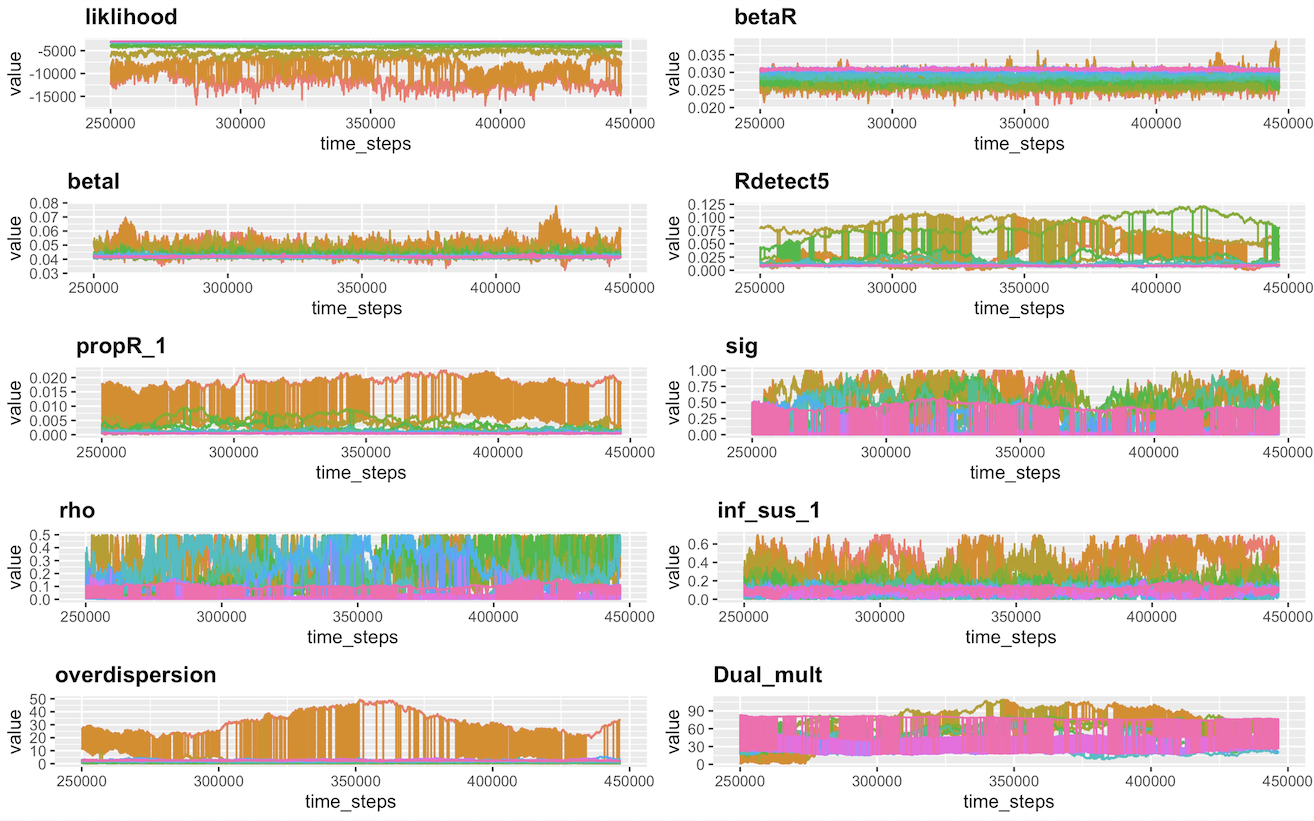


***Figure C: Parallel Tempering Trace.*** *Sample trace from parallel tempering, showing a subsection of parameters. Each colour is a chain at a different temperature, where the main chain is pink and the chain at the highest temperatures is red. The chains are thinned to 1:10.*


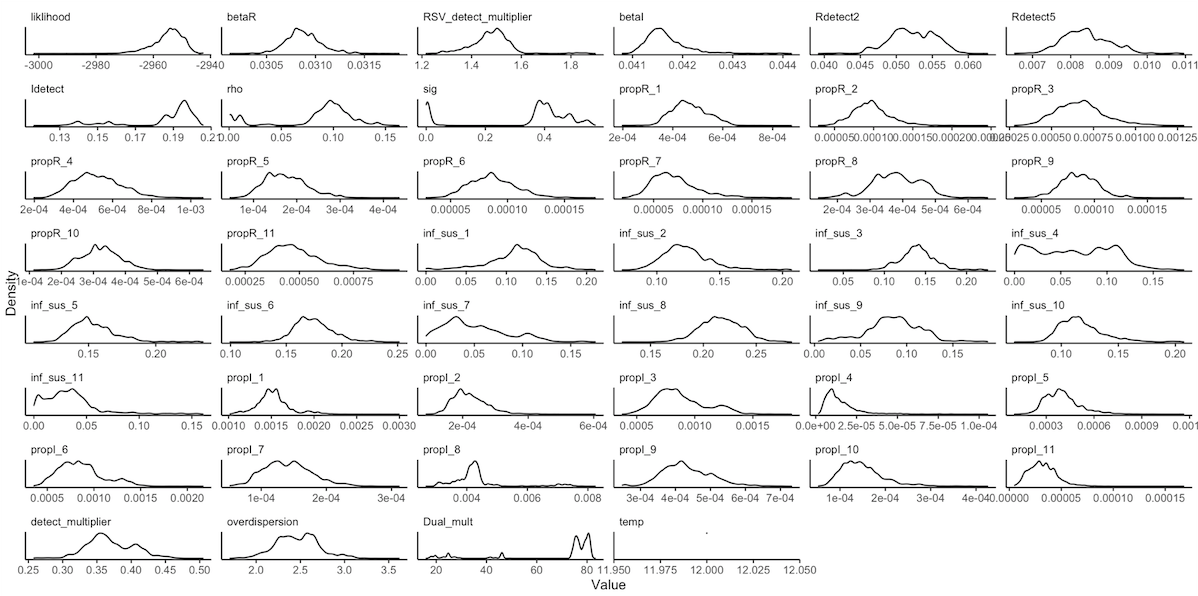


***Figure D: Posterior Density.*** *Density of fitted parameters from the final sample.*


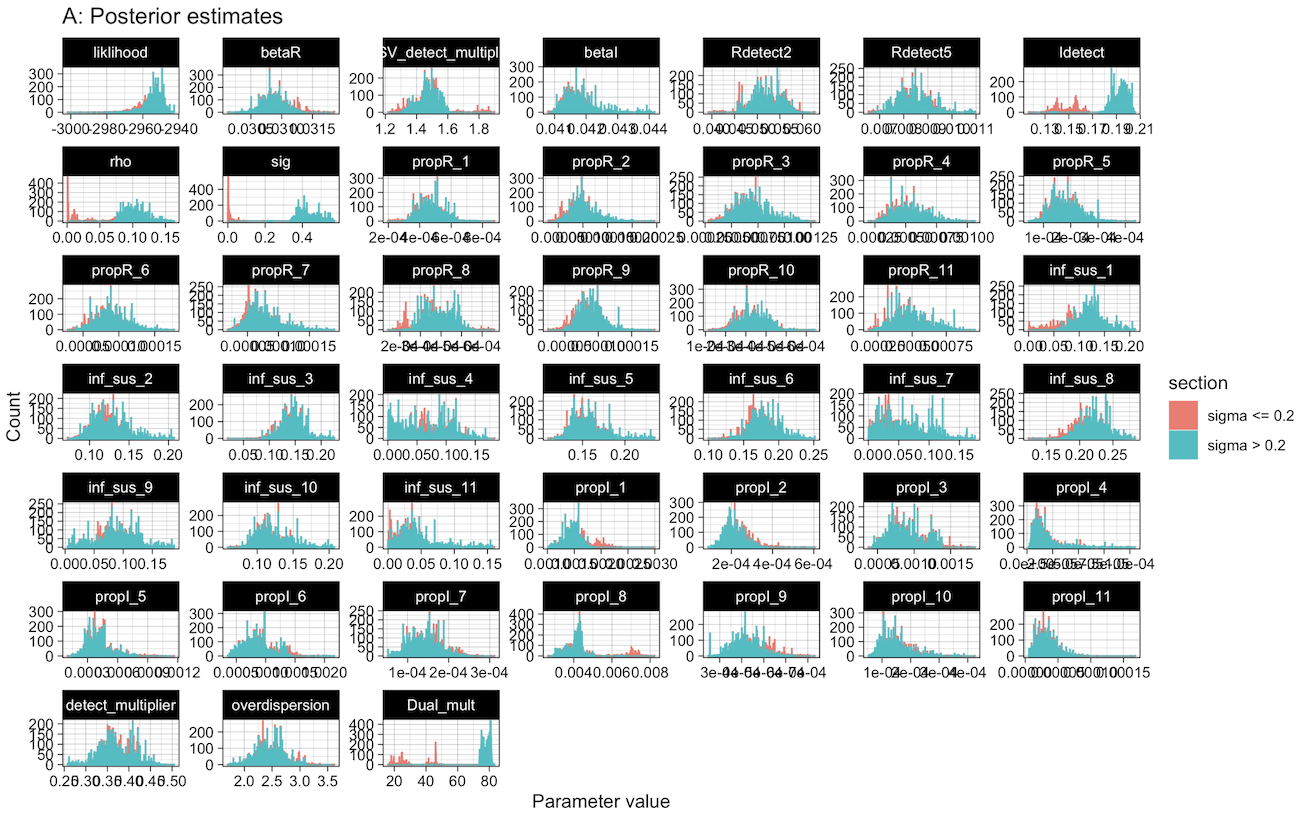
***Figure E: Posterior parameter estimates, split by the value of sigma (interaction parameter)***

## Attack Rates & Circulation patterns

Figure F shows the Attack Rates for each virus, season and age group, as well as the susceptibility to influenza at the start of the season by age group, calculated from 50 posterior samples.

We estimated a seasonal attack rate ranging from 24% to 41% for RSV and 1% to 15% for Influenza. For RSV, the attack rate was lowest in the oldest age group of 65+, whereas for influenza the lowest attack rates were in the youngest age group of 0-1 years old. Susceptibility to influenza at the start of the season was high, with all age groups in all years being over 87% susceptible to infection with the circulating strain.


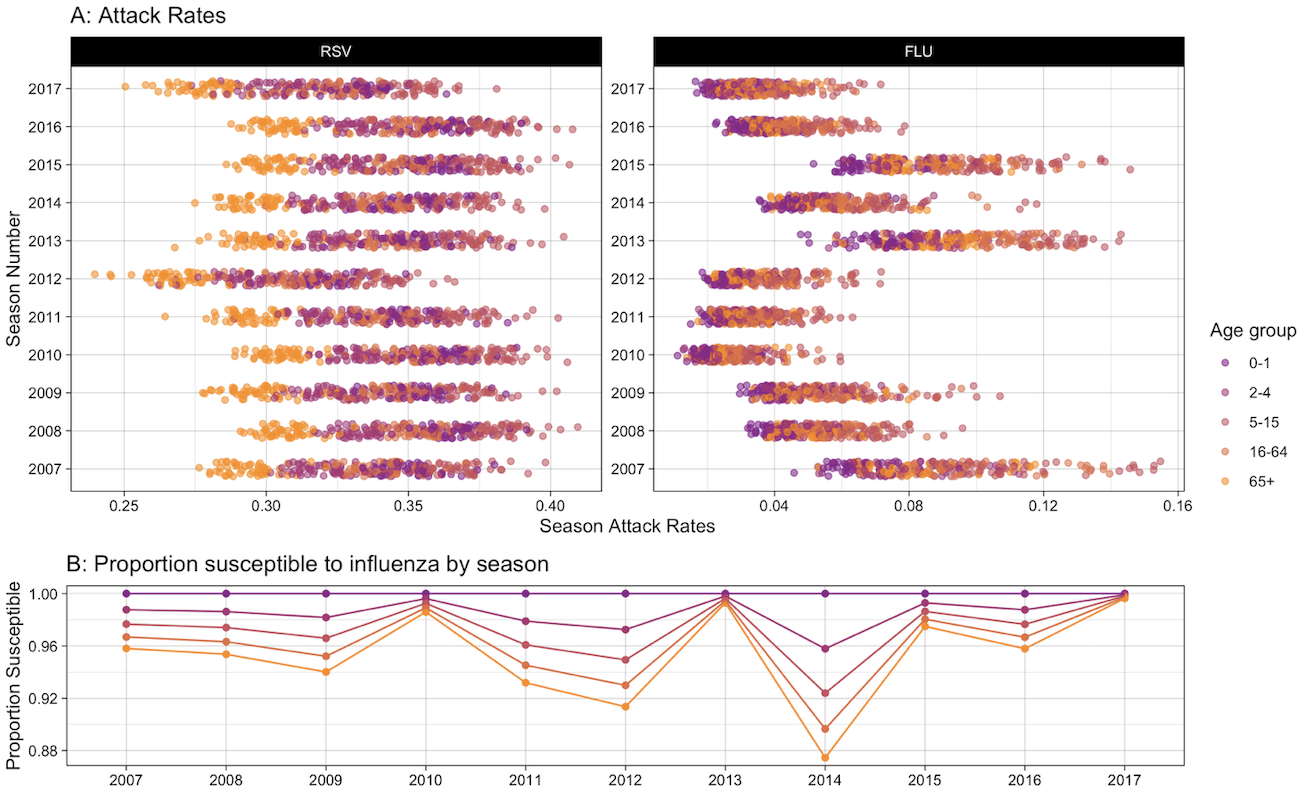


***Figure F: Modelled Output.*** *A) Season attack rates for influenza and RSV by age group. B) Proportion susceptible to influenza at the beginning of the season for each year by age group, using the median value of the posterior samples. Each year the susceptibility to infection of each age group is defined by one parameter in an exponential function, see supplement section 6 for details.*

Figure G shows the weekly incidence of infections across all age groups and Figure H shows the weekly incidence per 100’000 population of the age group over time. Age group 5-15 has the highest incidence proportion peak across both viruses.

*
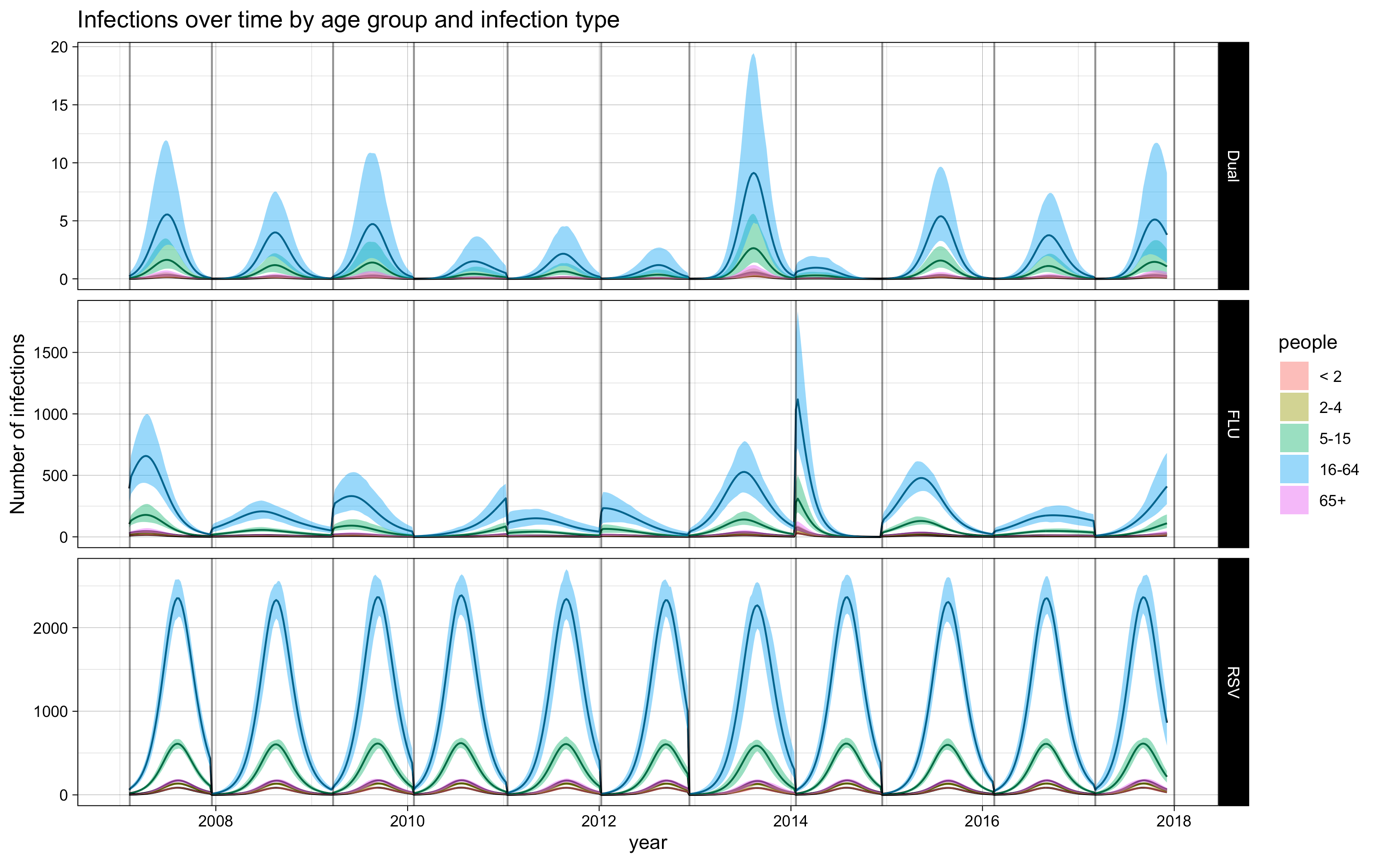
* ***Figure G: Modelled Incidence.*** *Weekly number of incident infections over time, for each virus type and age group. The line indicates the median value across 100 samples, and the ribbon the 95% CrIs.*

*
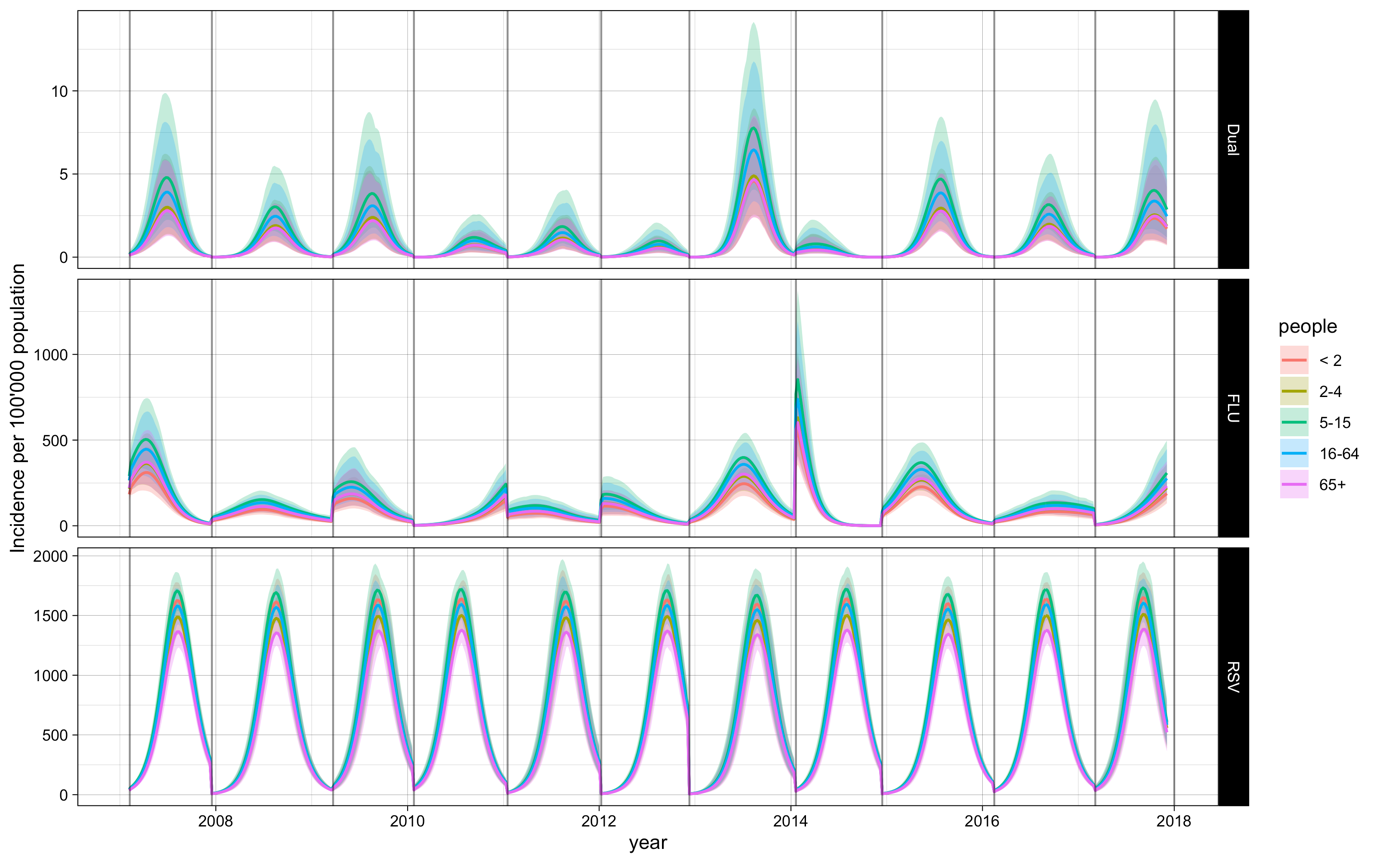
*

***Figure H: Modeled weekly Incidence per 100’000 population.*** *Median infection incidence proportion over time, for each virus type and age group, with 95% quantiles shown in the ribbon.*

## Mode-specific model fits and profile likelihoods

The log likelihoods between the modes do not show significant differences, with the median value of -2954.7 (95%CrI: -2970.5 - - 2945.8) in the low interaction mode and -2953.5 (95%CrI: -2964.0 - -2946.7) in the moderate interaction mode. There are no visible differences in the model fit (shown for each mode in Figures I and J).


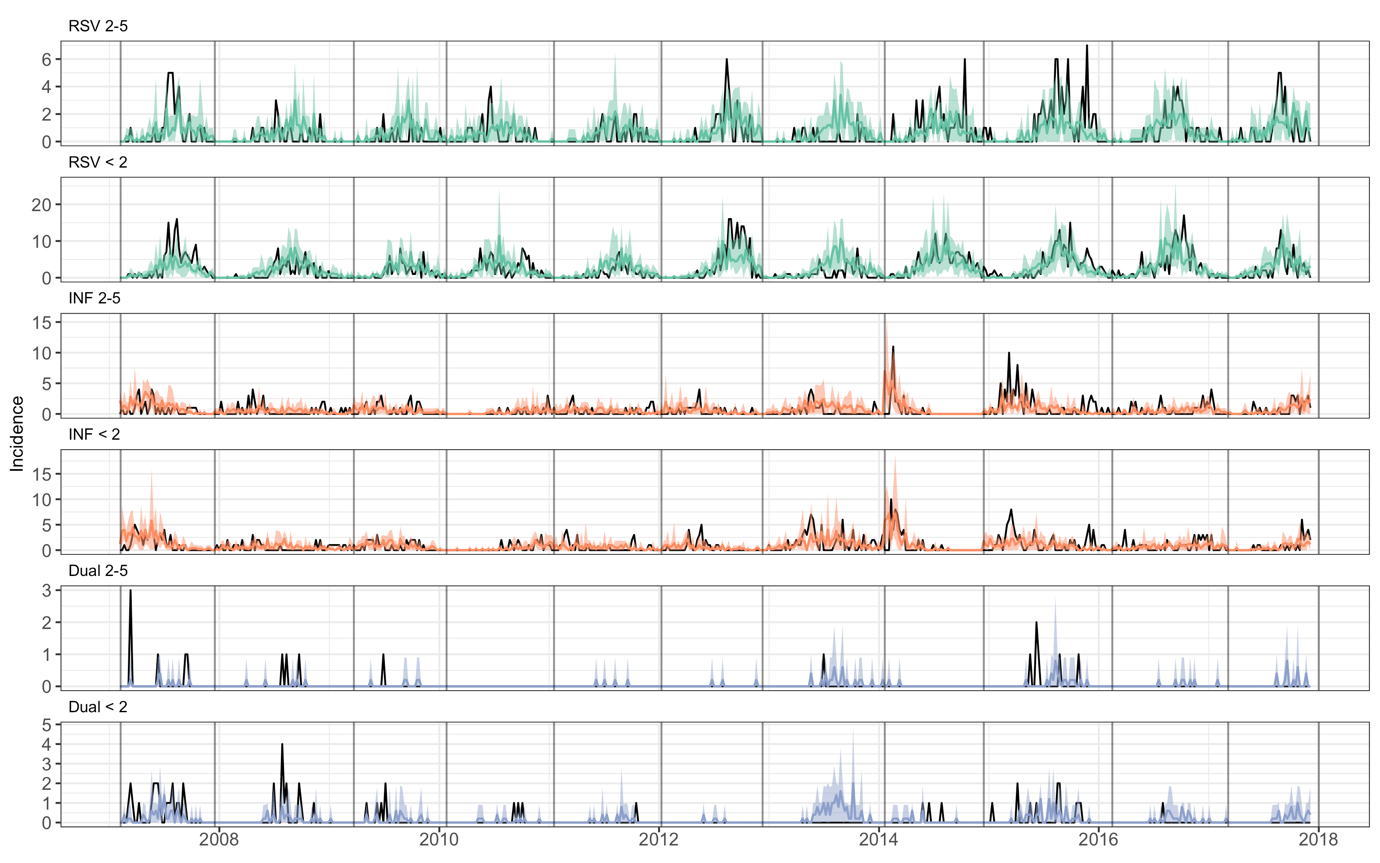
 ***Figure I: Model Fit at the low interaction mode****: Black lines are the data, coloured lines are the 95% CrI posterior predictive interval and the mean. Panels show the fit by age group and Virus, and samples were included that had a value for the iinteraction parameter lower than 0.2.*
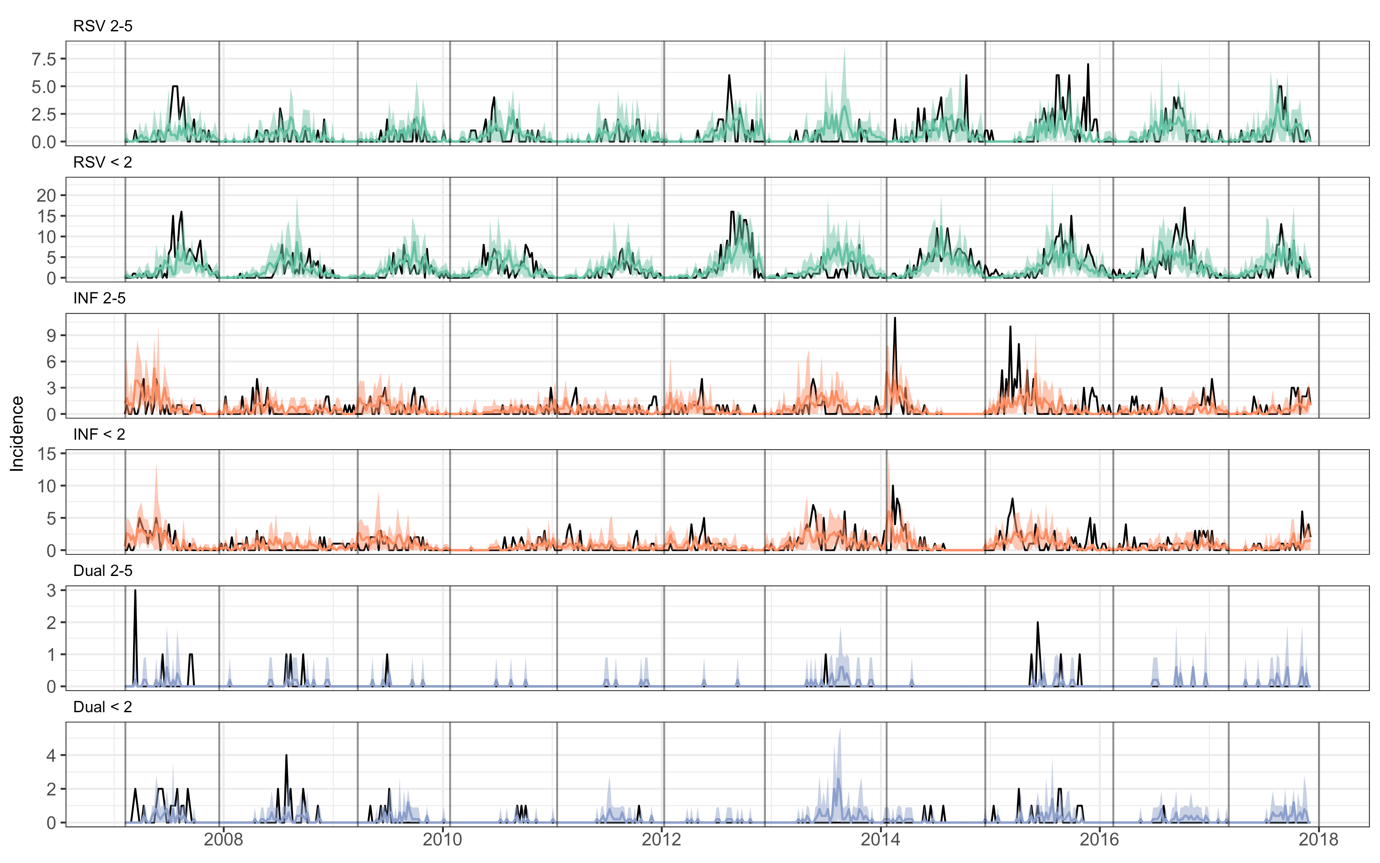
 ***Figure J: Model Fit at the moderate interaction mode****: Black lines are the data, coloured lines are the 95% CrI posterior predictive interval and the mean. Panels show the fit by age group and Virus, and samples were included that had a value for the iinteraction parameter higher than 0.2*

Figure K shows the profile likelihoods of the interaction parameter, at the two different modes. Samples were taken from the posterior from each mode, and the value of the interaction parameter was then changed to values between 0 and 1, with an interval of 0.02. The log likelihood of each of these points was calculated, and the median and 95% credible interval was then plotted. Infinite values of the log likelihood were excluded from the calculation of the median and credible interval.


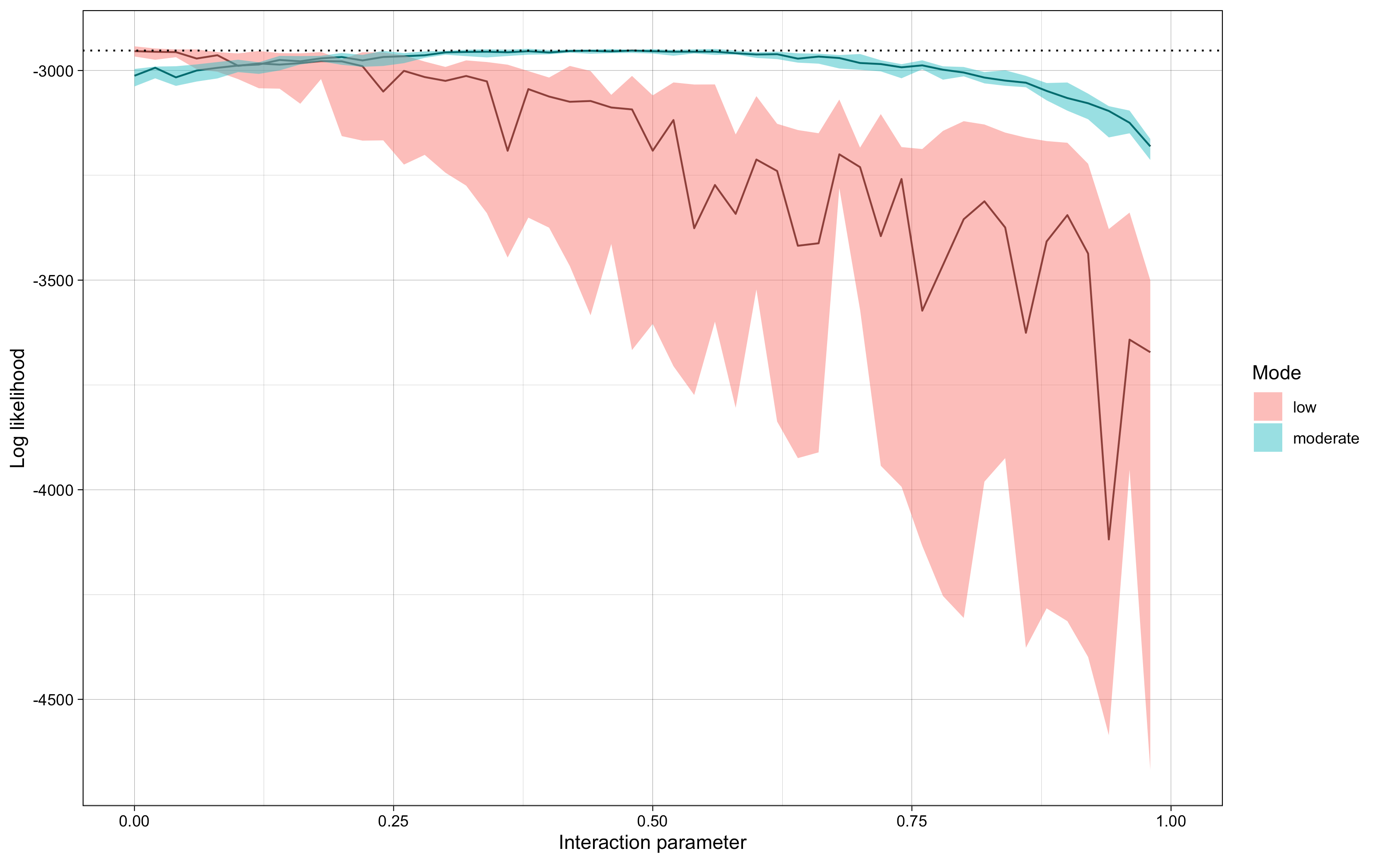


***Figure K: Profile likelihoods of the interaction parameter:*** *Lines indicate the median and ribbons the 95% CrI by mode. The dotted line is placed at the maximum value of the median across both modes.*

## Sensitivity to severity of dual infected cases

We tested the assumption of the severity of dual infected cases, by rerunning the fit without the parameter that multiplied the proportion of RSV detected to give a new dual infection detection rate. Instead the dual infections had the same reporting rate as for RSV. This set of chains were run for 100000 iterations and 50000 was discarded as burnin, and then the remaining samples were thinned to 1 in 10. Cross-protection estimates overlapped with estimates of the ‘no interaction’ mode in the main model, with the posterior for interaction at 0.008 (95%Ci 0.00 - 0.04) compared to 0.004 (95%CI 0.000 - 0.046).


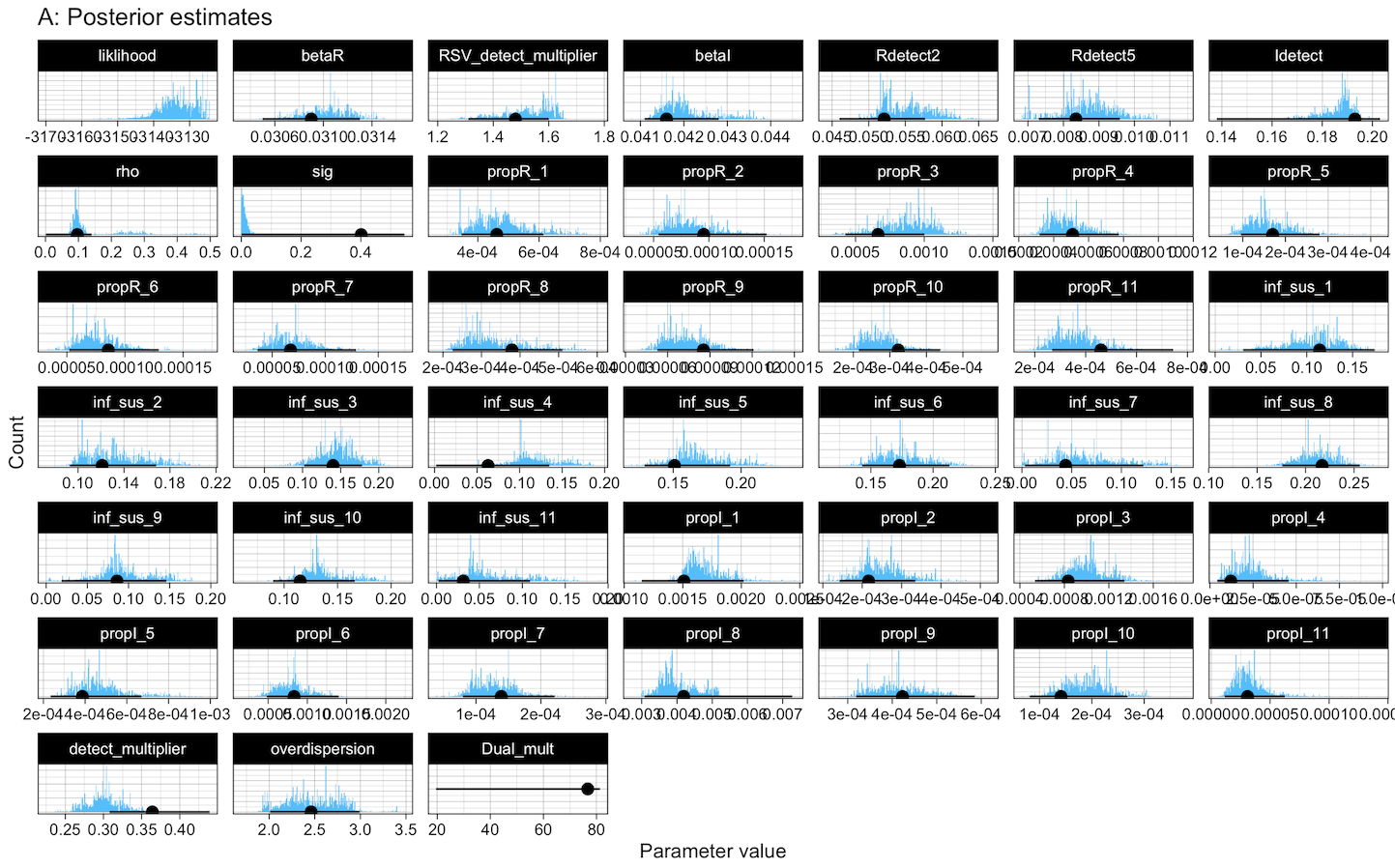


***Figure L: Parameter Density.*** *Density of fitted parameters from the final sample. Black lines show the median and 95%CI for the main model run.*

## Prior Sensitivity

As a sensitivity analysis, we reran the model fit with a prior for a high strength of interaction (normal distribution, mean = 0.8, standard deviation = 0.15). This is due to the existing evidence of cross-protection. Figure M shows the posterior estimates for the parameters. This set of chains were run for 100000 iterations and 25000 was discarded as burnin, and then the remaining samples were thinned to 1 in 10. Cross-protection estimates overlapped with estimates of the ‘moderate interaction’ mode in the main model, with the posterior for interaction at 0.22 (95%Ci 0.13 - 0.47) compared to 0.41 (95% 0.36 - 0.54) and the duration of cross-protection at 5.2 days (95%CI 3.1 -10) compared to 10.0 days (95%CI 7.1 -12.8 days).


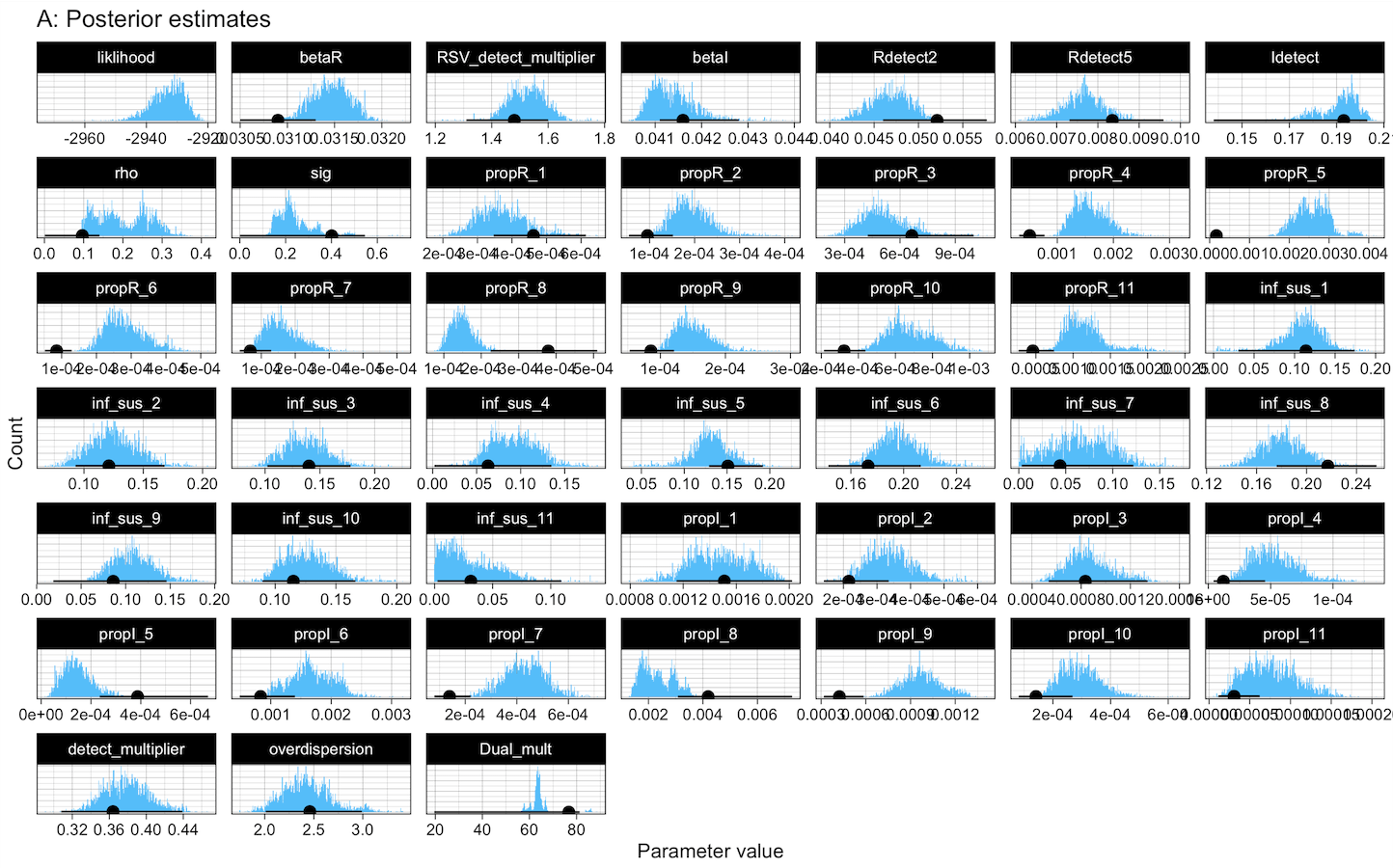


***Figure M: Parameter Density with interaction prior.*** *Density of fitted parameters from the model with a prior for strong cross-protection.*

## Sensitivity on interaction direction

In the main manuscript model, the estimated cross-protection due to reduced susceptibility to secondary infection is assumed to be bi-directional. This is because the proposed mechanism of innate immunity activation would be unlikely to differ, depending on the direction of infection. However, as the influenza epidemic typically precedes the RSV epidemic in this setting, the estimate is mostly based on the cross-protection of influenza on RSV. To demonstrate this, we ran simulations from posteri parameter estimates but with either no or complete cross-protection of RSV infection against subsequent flu infection. The main difference here is the number of dual infections reported, but it does not have a strong influence on the epidemiology of both viruses.

*
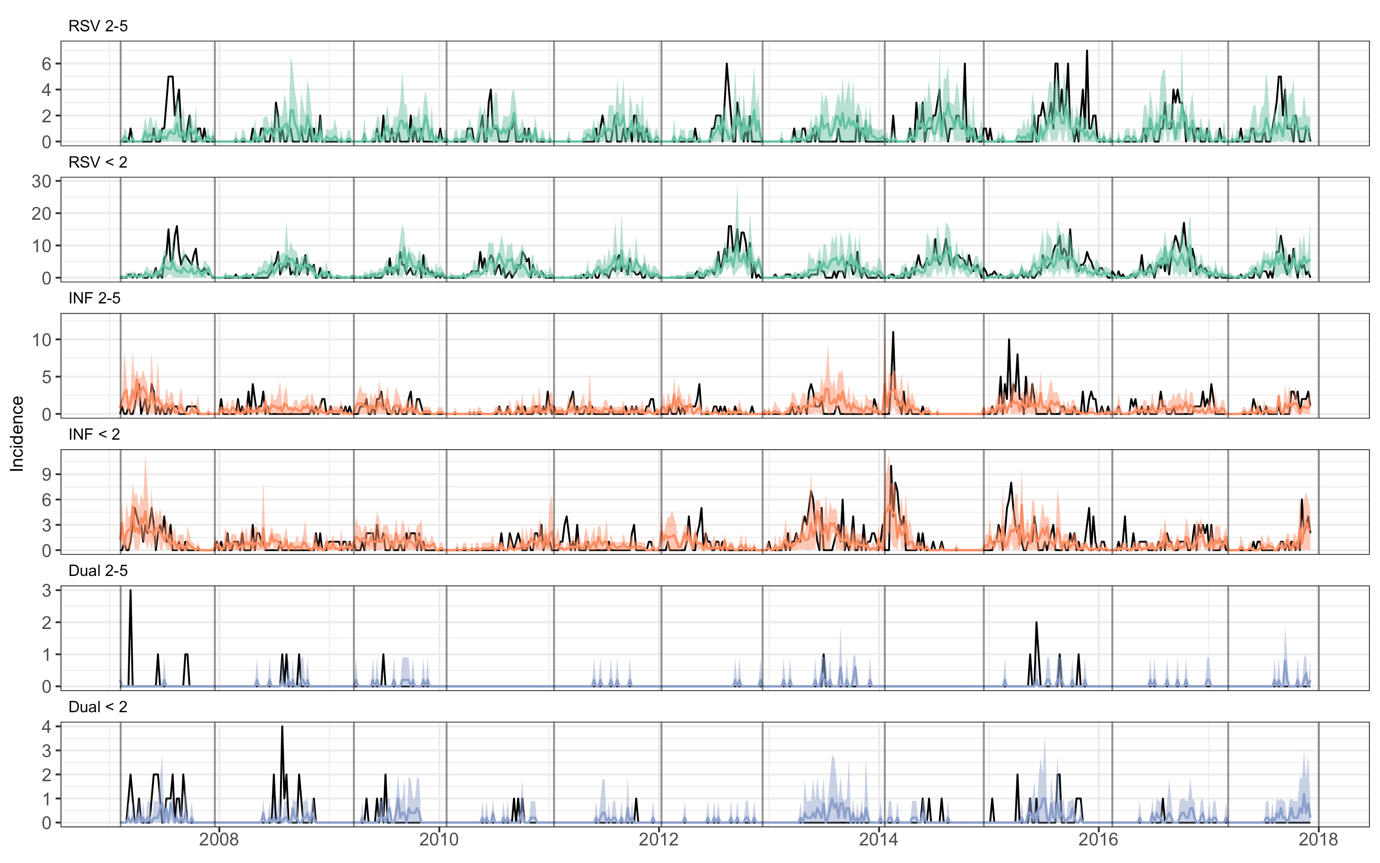
* ***Figure N: Simulations with uni-directionaly cross-protection.*** *Black lines are the data, coloured lines are the 95% CrI posterior predictive interval and the mean. Panels show the fit by age group and Virus. Cross-protection of RSV on influenza susceptibility was fixed at 1, complete cross-protection*

*
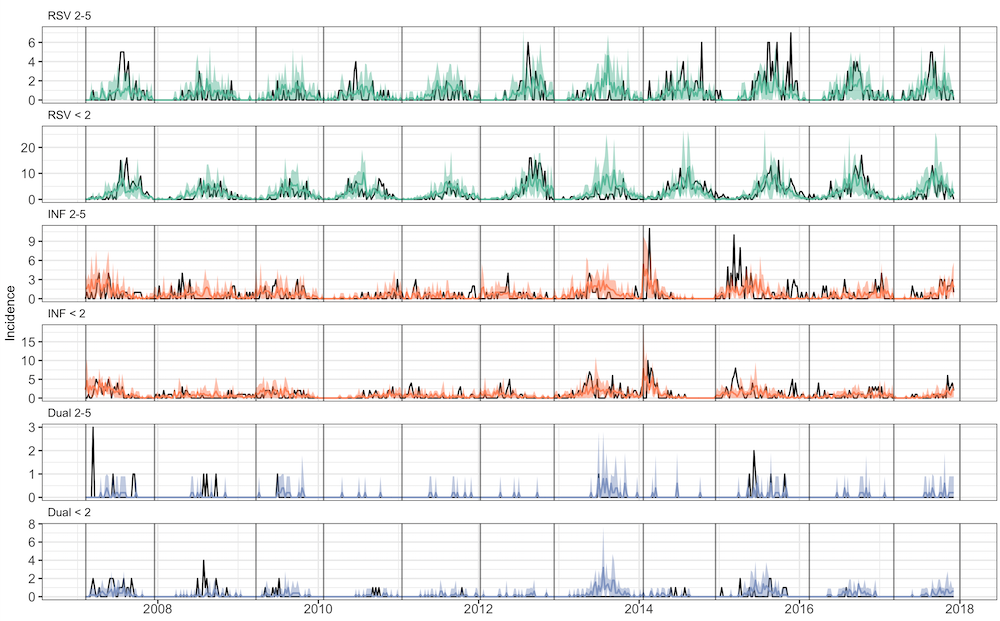
*

***Figure O: Simulations with uni-directionaly cross-protection.*** *Black lines are the data, coloured lines are the 95% CrI posterior predictive interval and the mean. Panels show the fit by age group and Virus. Cross-protection of RSV on influenza susceptibility was fixed at 0, no cross-protection*

## References

1. Diekmann, O., Heesterbeek, J. A. P. & Roberts, M. G. The construction of next-generation matrices for compartmental epidemic models. *J. R. Soc. Interface* **7**, 873–85 (2010).

2. Henderson, F. W., Collier, A. M., Clyde, W. A. & Denny, F. W. Respiratory-Syncytial-Virus Infections, Reinfections and Immunity. *N. Engl. J. Med.* **300**, 530–534 (1979).

3. Glezen, W. P., Paredes, A. & Taber, L. H. Influenza in Children. *JAMA* **243**, 1345–1345 (1980).
