# Supplementary material for: Evidence for influenza and RSV interaction from 10 years of enhanced surveillance in Nha Trang, Vietnam, a modelling study
Source: PLoS Comput Biol. 2022 Jun 24;18(6):e1010234. doi: 10.1371/journal.pcbi.1010234 (PMC9262224; doi:10.1371/journal.pcbi.1010234)
Supplement: Attachment — Submitted filename: NhaTrang Reviewer response.pdf [file pcbi.1010234.s002.pdf]

Dear Editor,

Thank you for the helpful comments and reviews. We have addressed all the points raised and clarified any inconsistencies. These changes have improved the manuscript. We hope that the manuscript is now acceptable for publication in *PLOS Computational Biology*.

Yours Sincerely

Naomi Waterlow

Reviewer #1: Overall, this is a well-done and clearly-written analysis of interactions between Influenza and RSV using detailed surveillance data from Vietnam. The authors developed an age-stratified transmission model for RSV and influenza, which was fitted to children aged <5 years with either one or two respiratory viruses infections to determine the strength of cross-protection and the effects of co-infections on disease severity. The authors concluded that co-infections increase the probability of being hospitalized and had a bimodal conclusion on cross-protection. The methods employed are well-justified and clearly articulated, and the conclusions appear to be broadly consistent with the results presented. However, I do have a few major comments regarding the methodology.

We would like to thank the reviewer for their comments and suggestions. They greatly improve the clarity of the manuscript. We have addressed each of the comments below.

[1] The authors fitted 15 parameters for each season individually (line 159 and table 1). The maximum time period for each season in the model is 66 weeks and it seems that RSV infections are mostly concentrated between November and May (line 172-line 173 and Figure 1C), which are about 26 weeks. I am concerned that the sparsity of the data may cause issues of parameter identifiability. I find it hard to be convinced that the whole model converged well giving that several parameters showed bimodal posterior distributions. I would recommend the authors use the entire 10 year period with yearly varying parameters of influenza and use weakly informative priors based on published literature, especially for those 0-inf priors.

We thank the reviewer for their comments. In terms of the bimodal posterior, we have taken a stringent approach and tried many methods to narrow down on the parameters space. We used a very robust inference technique (parallel tempering) in order to do this, and this technique is what enabled us to identify the bimodal posterior, as opposed to many more commonly used inference techniques such as MCMC, which would only have identified a single local minima. We have examined all the helpful suggestions from both reviewers 1 and 2, and following this remain convinced that the data does indeed support both parameter spaces, and that the only data able to discriminate between them would be very precise estimates of the proportion of infections that are detected by the ARI surveillance. The rough estimate that we have is in line with both modes (see below).

In addition to the responses to reviewer 1's suggestions, we have also done further analysis on the bimodal posterior based on the comments from reviewer 2. These include investigating more flexible forms of interaction that had the potential to explain the bimodal posteriors, a more in-depth view of the simulations in each mode and profile likelihoods on the interaction parameter. Please see the responses to reviewer 2 below for more details.

Regarding the specific point mentioned here, the model is currently fit to the entire 10 year period. By "fit each season individually" we mean that we do not track immunity over multiple years, but rather fit the susceptibility parameters at the start of each season. The reason for this is to avoid complexities as a result of immune cross-reactions between different subtypes of influenza and RSV. However, all parameters are fit in the same inference process, with key parameters such as the transmission rate and the interaction parameters fit across the entire time series simultaneously.

We thank the reviewer for noting this lack of clarity in the manuscript, and have updated it as follows:

> *<< We model the persisting immunity at the start of each season individually, with an initial proportion infected with each virus. This allows for a different immunity profile at the start of each season, without needing to account for waning of immunity and dynamics across virus subtypes specifically. For RSV we assume the only immunity at the beginning of the season is the age-specific reduction in susceptibility (leaky immunity), as immunity to reinfection typically lasts less than a year (25). Due to infections in previous influenza seasons and potential vaccination, susceptibility to influenza is assumed to decline exponentially at rate with age (see supplement section 6). >>*

Regarding changing the prior values, the vast majority of parameter posteriors do not have two clearly defined peaks, without high levels of overlap. The only ones that do are the two interaction parameters (strength and duration), the dual infection reporting multiplier, the detection rate of influenza and the proportion infected with influenza at the start of season 8 (2014). Therefore the most informative additional information we could have to distinguish between modes is the proportion of influenza infections that result in hospitalization, as we have little information on the interaction parameters and dual reporting multiplier. However, we have been unable to identify a reasonable prior for the reporting rate of influenza in a similar setting. This is specifically also the case because the estimated value of this parameter in the two modes has the same order of magnitude, estimating a median value of 15.9% (95% CrI: 13.5 - 16.9%) in the low interaction mode and 19.5% (95% CrI: 18.3 - 20.3%). We therefore feel it is unreasonable to put a stronger prior on this parameter. We do however discuss this parameter in the discussion.

> *<<While most of the posterior estimates are reasonable, the reporting rate for influenza infections is high, between 13 and 21%, compared to estimated 12% from data in Yoshida et al. (2013). However, many milder cases (including outpatients) are included*

in the reports as they may seek healthcare at the hospital, thereby increasing the expected reporting rate in this context. The posterior for the detection rate of influenza is one of the few parameters that, like the strength of cross-protection parameter, is bimodal. However, these modes do not differ greatly, with the medians of the two priors only differing by 3.6%, and therefore not substantially different to help us distinguish between the two modes.>>

[2] In the Supplement, Model equations section, the outflow from line 68 -(1-sigma)*lambda_INFi*PS_i does not equal to the inflow in line 72 lambda*PS_i. Also, if the epsilon means introduction rate from external sources, it should not show up as outflows from the susceptible population. It would also help readers to understand the equations if the authors can specify the meaning of each compartment like what is done in main text 138-139 and the captions of Figure 2C.

We thank the reviewer for noticing this. The equations mentioned on lines 68/72 do not equal the inflow due to a typing error in the supplement. The input into line 72 should read the same as the output to slide 28 and this has been updated in the manuscript.

For epsilon, we assume that the infection occurs as a result of a current member of the population being infected from an external source, rather than an introduction of a new infectious individual from an external source. We make this assumption in order to keep the population size constant. We have changed the definition of epsilon from “introduction” from external source to “infection” from external source.

We have added extra information on the compartment meanings and transmission parameters as requested:

<< Full model equations are shown below. Each compartment includes the state for both RSV and influenza, with the first letter indicating the state for RSV, and the second for influenza. E.g. is shorthand for . Subscripts used are “INF” for influenza and “RSV”. Susceptibles become infected at force of infections λ_{INF} and λ_{RSV} , and move into the I states. They then remain infectious for $1/\gamma_{INF}$ and $1/\gamma_{RSV}$ days and during the infectious period and $1/\rho$ days thereafter they are cross-protected and thus their propensity for heterologous infection is reduced by factor σ, the strength of cross-protection. All age groups are equally susceptible to influenza, but there is reduced susceptibility to RSV in older age groups, determined by parameter τ_i. >>

Minor comments:

Introduction

[3] It may be better to clarify what is the “interaction” that this paper refers to. In lines 88 and 89, “to estimate the strength of cross-protection and the effects of co-infections on disease severity” may be more precise as it corresponds to the conclusions.

We thank the reviewer for the suggested wording, it has now been updated to this in the manuscript.

Methods

[4] Line 98: Do you have information on the 6% that were not enrolled in the study? Are their demographic background (remote living? who may have less chance of interactions with those living in the city) and contact patterns (such as household size) the same as those enrolled in the study?

Unfortunately, we do not have information on the participants that were not enrolled in the study. However, we think you raise an important point, so have added a discussion on this as follows:

> *<< [...] This study also has a high participation rate, however we do not have information on participants who declined to take part in the study, and this may bias the results. For example, there may be a severity bias where very sick children are less likely to be enrolled, which could lead to a slight underestimate of the percentage dual infected. >>*

[5] Line 100: Can you provide the sensitivity and specificity of the multiplex-PCR test?

The detection limits of the multiplex PCR assay used for influenza and RSV detection were ~10 copies/ul (10-100 copies/reaction). However, any sensitivity would get absorbed into the reporting rates for the viruses.

[6] Line 198: Please provide information on how many interactions were left for the parameter estimates.

This has been added as requested:

> *<<We ran the parallel tempering algorithm with 12 chains and 450,000 iterations. The initial 250,000 iterations were discarded as burn-in. Accepted samples from the first chain were then thinned to 1 in 10 for analysis, resulting in a final sample size of 20’000. >>*

Reviewer #2:

The authors aim to better quantify the interaction between influenza virus and RSV, using data on pediatric infection from Nha Trang in Vietnam, and using a transmission modeling approach.

I appreciate the challenging nature of this type of study. But in this current form, I find the study lacking in several ways, and I have tried to describe these below. Perhaps some of these shortcomings can be addressed, in which case, it could be a worthwhile contribution to this field.

We thank the reviewer for their helpful comments and suggestions. We have addressed all the points in turn below.

In terms of the bimodal posterior, we have taken a stringent approach and tried many methods to narrow down on the parameters space. We used a very robust inference technique (parallel tempering) in order to do this, and this technique is what enabled us to identify the bimodal posterior, as opposed to many more commonly used inference techniques such as MCMC, which would only have identified a single local minima. We have examined all the helpful suggestions from both reviewers 1 and 2, and following this remain convinced that the data does indeed support both parameter spaces, and that the only data to discriminate would be very precise estimates of the proportion of infections that are detected by the ARI surveillance. The rough estimate that we have is in line with both modes (see below).

1. Important parts of the transmission model (and data) are not presented or adequately described.

It is unclear what assumptions the authors make about the dynamics of and transmission from individuals >5 years? It is stated in the methods that older age groups are modeled (which is good), but the data used to calibrate these, and the results (ie, incidence of flu and RSV from model simulations) are lacking.

Given that >5 population will be substantially larger, and may play an overriding role in driving the dynamics of pediatric infections, it should be described with more clarity and supported with data.

Similarly, it is unclear if and how seasonality is modeled. Given seasonal nature of transmission, especially of RSV, I think it would be important to carefully include this.

We thank the reviewer for this comment and agree that including more detail on the circulation of the viruses in age groups >5 is informative and have therefore added this to the supplement. The model was fit using data in the under 5 population, not the older populations, as this data was unavailable. However, the model used age-specific contact rates and susceptibilities as input into the dynamic model.

> <<
> *Attack Rates & Circulation patterns*
>
> *Figure S5 shows the Attack Rates for each virus, season and age group, as well as the susceptibility to influenza at the start of the season by age group, calculated from 50 posterior samples.*
>
> *We estimated a seasonal attack rate ranging from 24% to 41% for RSV and 1% to 15% for Influenza. For RSV, the attack rate was lowest in the oldest age group of 65+, whereas for influenza the lowest attack rates were in the youngest age group of 0-1 years old. Susceptibility to influenza at the start of the season was high, with all age groups in all years being over 87% susceptible to infection with the circulating strain.*

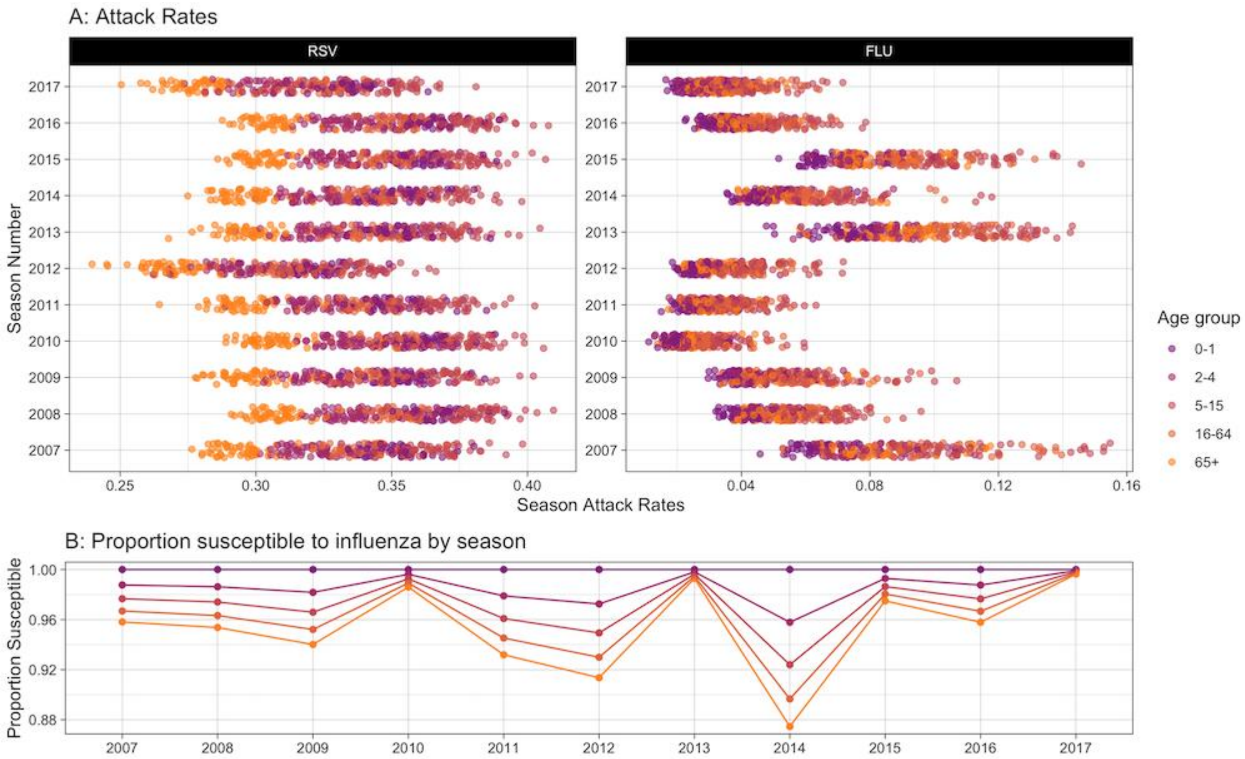

Figure S6: Modelled Output. *A) Season attack rates for influenza and RSV by age group. B) Proportion susceptible to influenza at the beginning of the season for each year by age group, using the median value of the posterior samples. Each year the susceptibility to infection of each age group is defined by one parameter in an exponential function, see supplement section 6 for details.*

Figure S7 shows the weekly incidence of infections across all age groups and Figure S8 shows the weekly incidence per 100'000 population of the age group over time. Age group 5-15 has the highest incidence proportion peak across both viruses.

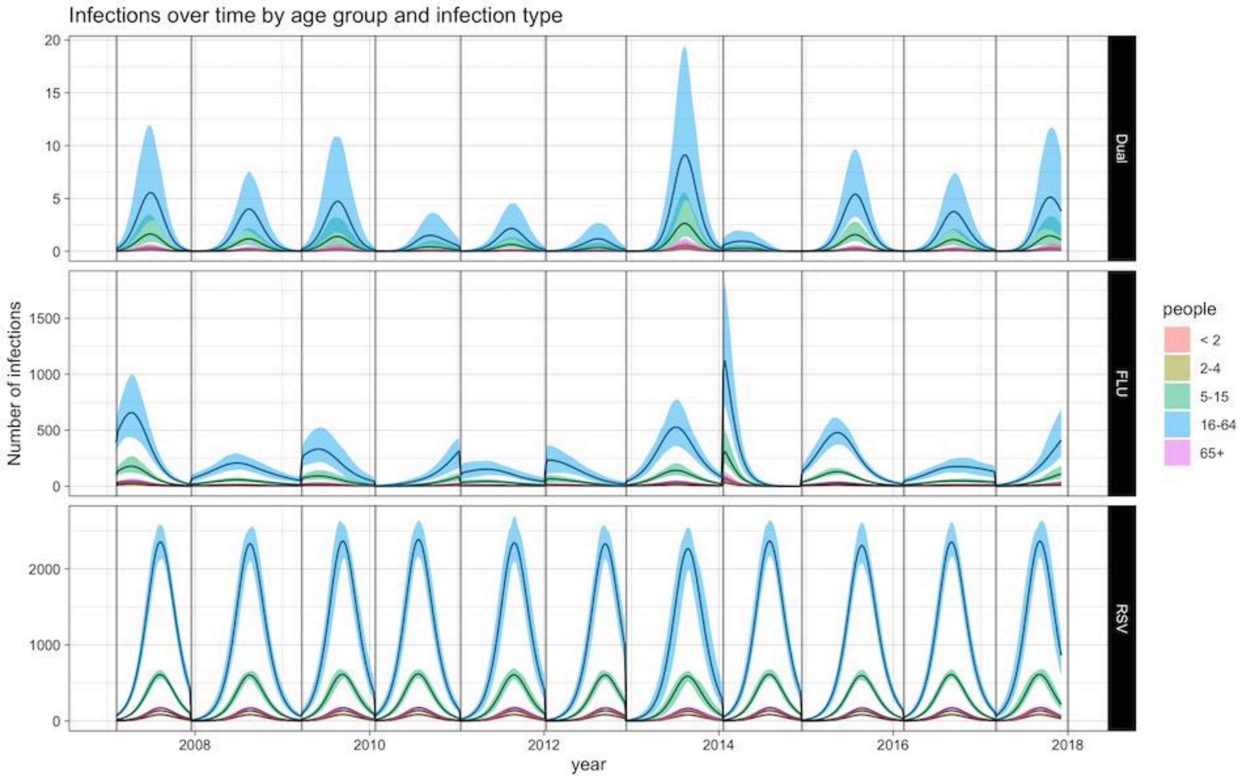

Figure S7: Modeled Incidence. *Weekly number of incident infections over time, for each virus type and age group. The line indicates the median value across 100 samples, and the ribbon the 95% CrIs.*

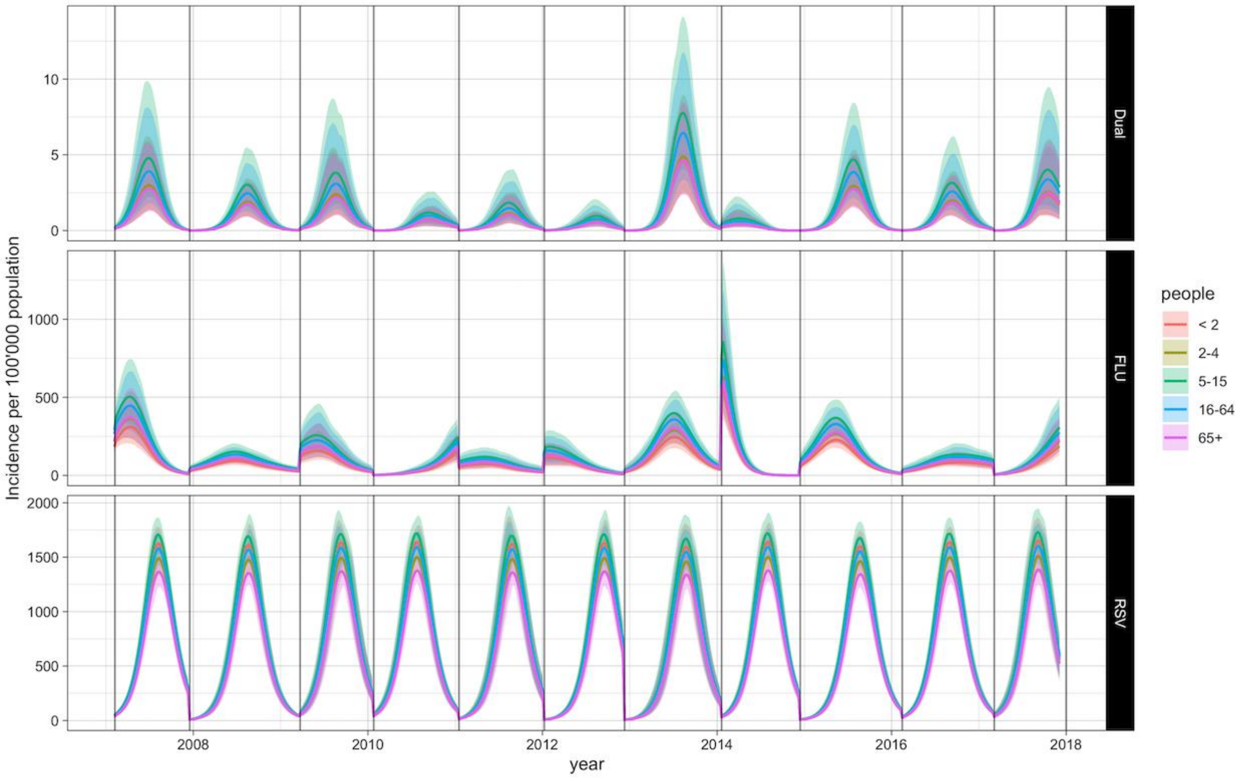

Figure S8: Modeled weekly Incidence per 100'000 population. *Median infection incidence proportion over time, for each virus type and age group, with 95% quantiles shown in the ribbon.*

>>

In terms of seasonality, it has not been directly included in the model. Instead, the proportion susceptible at the start of each season has been fit. This has been stated more clearly in the manuscript:

<<We model the persisting immunity at the start of each season individually, with an initial proportion infected with each virus. This allows for a different immunity profile at the start of each season, without needing to account for waning of immunity, dynamics across virus subtypes and seasonality specifically.>>

2. The model for interaction between influenza and RSV, is not conceptualized or described in detail.

In the background, authors describe that 1. increased severity of co-infections and 2. cross protection between two viral infections as the two pathways of interactions that the authors are interested in estimating.

However, it is unclear how severity of co-infections are modeled; and what data are used to measure severe forms of co-infections.

For example, cross protection is modeled in a specific way, and it is unclear to me that the assumptions are justified. For example, it is assumed to be symmetric -- that protection from an infection of influenza to RSV would be the same in the opposite direction?

The evidence that authors cite in the background are mostly protection from prior influenza infection on RSV. At the very least, the modeling framework should be flexible to allow for protective interaction in one direction.

The fact that they find bimodality in their results may also be pointing to this.

Relatedly, while the duration of the cross protection is explored, the onset of the cross-protection is assumed to begin instantaneously. It would be importantly explore the timing, especially given that some of the studies they site report protection after several weeks.

Our measure of severity is the propensity of an infection being "severe enough" to be reported; i.e. be admitted to the local hospital. This has been clarified in the manuscript as follows:

<< We use this data in combination with a dynamic transmission model to estimate the strength of cross-protection and the effects of co-infections on disease severity (defined as the proportion of infections that require hospital attendance).>>

In all but two years studied (in which two the influenza activity was very low) the influenza peak occurs before the RSV peak, so the level of interaction we have estimated mainly pertains to the impact of influenza on RSV susceptibility, not the other way around. To demonstrate this, we have run simulations with parameter values from our posterior, but replacing the cross protection induced by RSV infection on influenza susceptibility by either no or complete cross-protection. These simulations demonstrate that the main aspect we are estimating is the cross-protection from RSV on influenza infection, with the major impact of altering the influenza on RSV cross-protection on the number of dual infections. Given our interaction parameter therefore mainly estimates the impact of influenza on RSV, we do not think this is an explanation for the bimodality in the inference results.

Accordingly, we have added this sensitivity analysis to the supplement, and expanded the discussion.

Supplement:

<<

Sensitivity on interaction direction

In the main manuscript model, the estimated cross-protection due to reduced susceptibility to secondary infection is assumed to be bi-directional. This is because the proposed mechanism of innate immunity activation would be unlikely to differ, depending on the direction of infection. However, as the influenza epidemic typically precedes the RSV epidemic in this setting, the estimate is mostly based on the cross-protection of influenza on RSV. To demonstrate this, we ran simulations from posterori parameter estimates but with either no or complete cross-protection of RSV infection against subsequent influenza infection. The main difference here is the number of dual infections reported, but it does not have a strong influence on the epidemiology of both viruses.

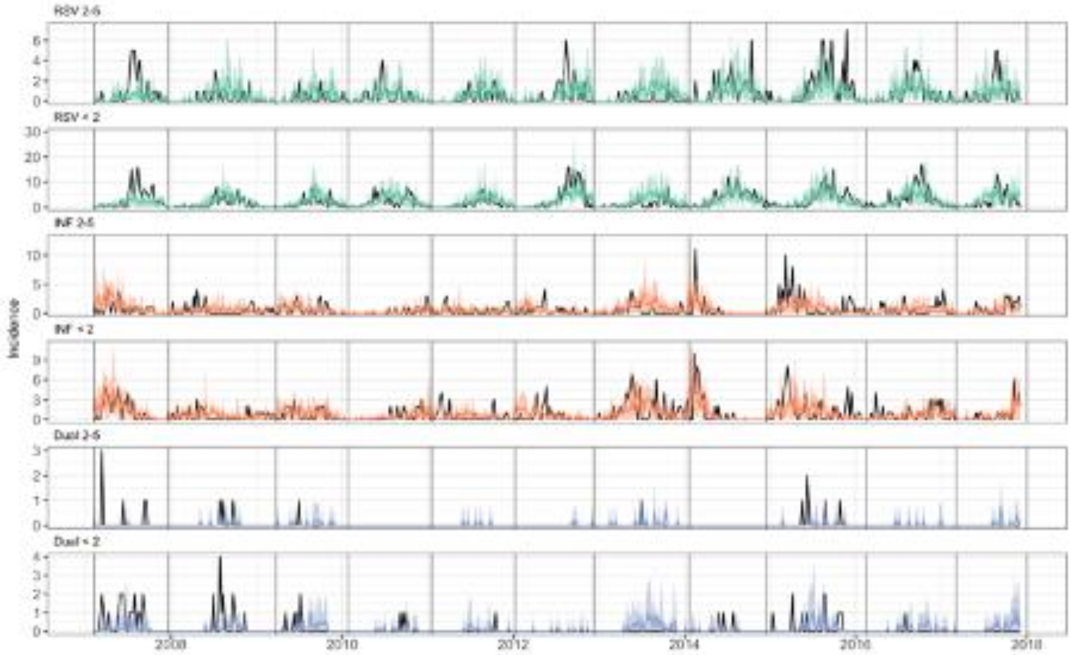

Figure S14: Simulations with uni-directionaly cross-protection. *Black lines are the data, coloured lines are the 95% CrI posterior predictive interval and the mean. Panels show the fit by age group and Virus. Cross-protection of RSV on influenza susceptibility was fixed at 1, complete cross-protection*

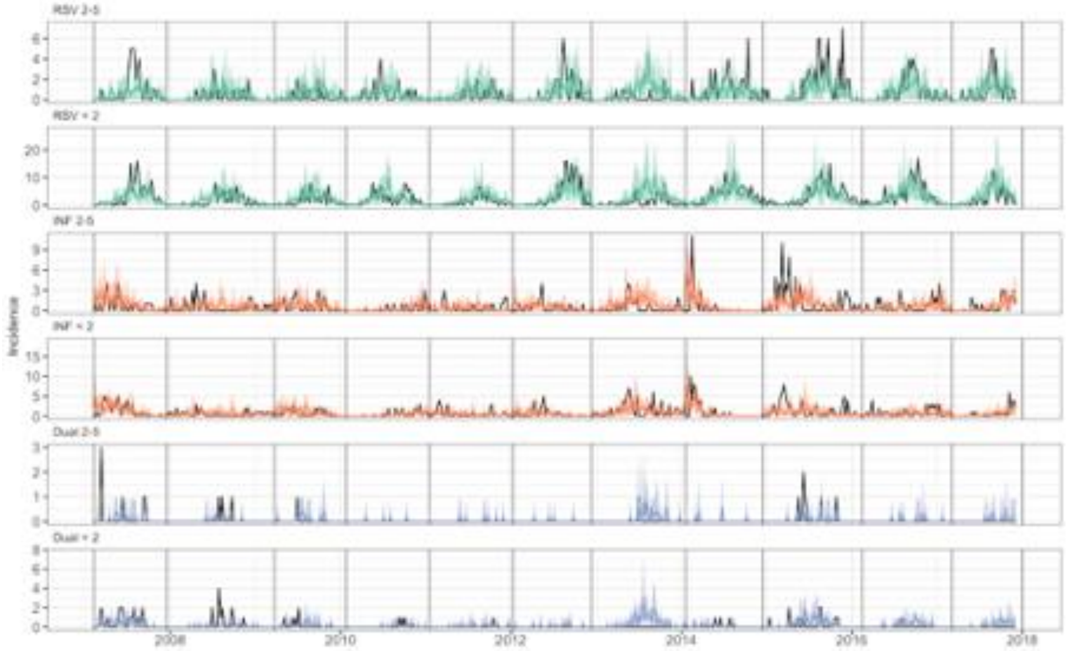

Figure S15: Simulations with uni-directionaly cross-protection. *Black lines are the data, coloured lines are the 95% CrI posterior predictive interval and the mean. Panels show the fit by age group and Virus. Cross-protection of RSV on influenza susceptibility was fixed at 0, no cross-protection*

>>

Discussion:

<< *In our model we assumed the cross-protection between the two viruses to be bi-directional, having the same impact irrelevant of which virus caused the first infection. This assumption is based on the mechanism of cross-protection being the activation of the innate immune system into a general antiviral state. However, in the study site the influenza epidemic occurs before the RSV epidemic, therefore our estimate of the strength of cross-protectionn on susceptibility to the second virus is mainly an estimate of the impact of influenza on RSV, and does not necessarily capture any dynamics in the other direction.* >>

We have explored the duration of cross-protection, defined by our parameter ρ. This parameter is fit as part of the inference process. As the proposed mechanism of interaction is activation of the innate immune system into an antiviral state, we assumed the interaction could occur upon first infection, as the innate immune system is activated upon infection. We have added to the discussion, recognizing that the model would not be able to capture such lags in infection:

<< *In addition, our model is not able to capture delays in the timing of cross-protection, which could potentially occur when considering other mechanisms of cross-protection.* >>

3. I find it hard to make sense of the findings -- 41% cross-protection for 10 days or nothing. I can make sense of a finding where the data and the model are not able to find statistically significant evidence of interaction -- that no interaction and some interactions are equally likely.

But from what the authors are presenting, it appears to argue for two very distinct, yet very clearly defined interactions, which seems to be contradictory. I think this aspect needs to be explored in greater detail.

A few options may be:

(i) exploring more flexible forms of interaction (eg, allowing for asymmetry or delay in onset, as I have argued in 2)

We have investigated more flexible interaction as suggested here. See point 2 above for the response.

(ii) showing simulations of two explanations -- whether one model captures certain features of the data better.

We thank the reviewer for this idea - we have added the simulations as suggested to the supplement as follows:

<<

Mode-specific model fits

The log likelihoods between the modes do not show significant differences, with the median value of -2954.7 (95%CrI: -2970.5 - - 2945.8) in the low interaction mode and -2953.5 (95%CrI: -2964.0 - -2946.7) in the moderate interaction mode. There are no visible differences in the model fit (shown for each mode in Figures S9 and S10).

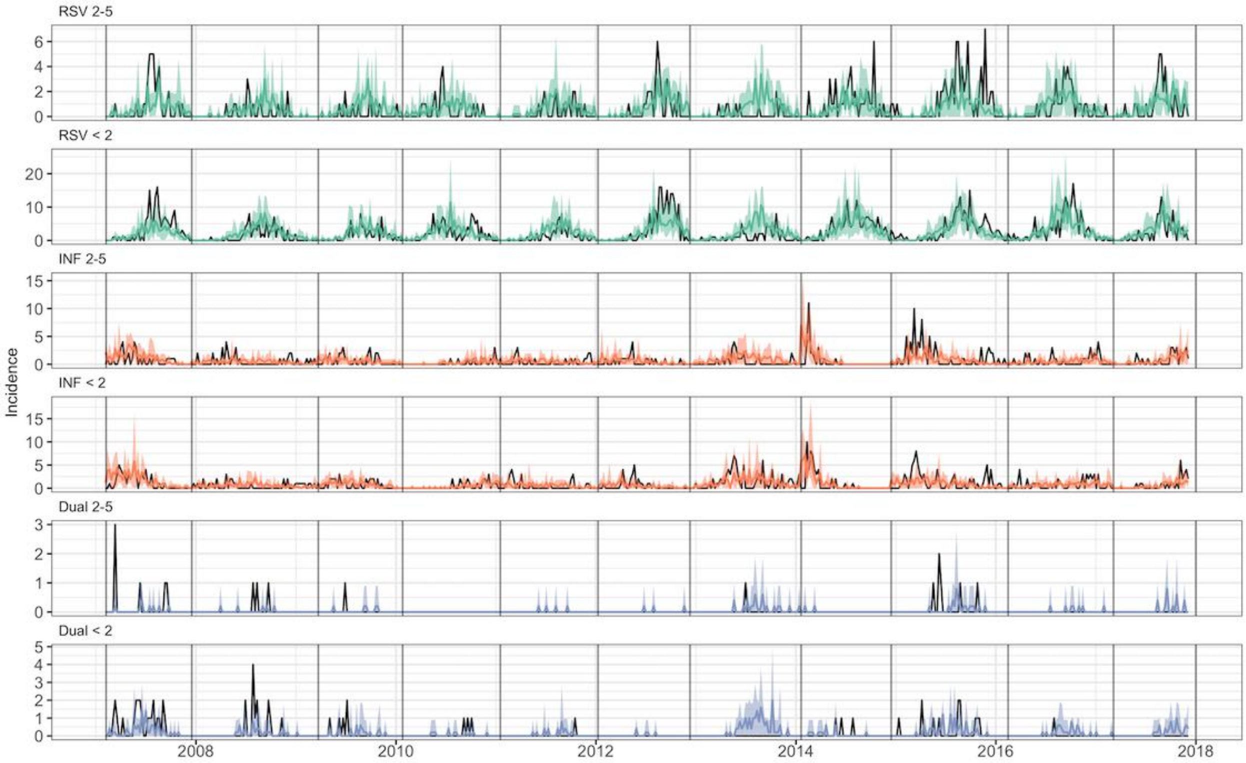

Figure S9: Model Fit at the low interaction mode: Black lines are the data, coloured lines are the 95% CrI posterior predictive interval and the mean. Panels show the fit by age group and Virus, and samples were included that had a value for the interaction parameter lower than 0.2.

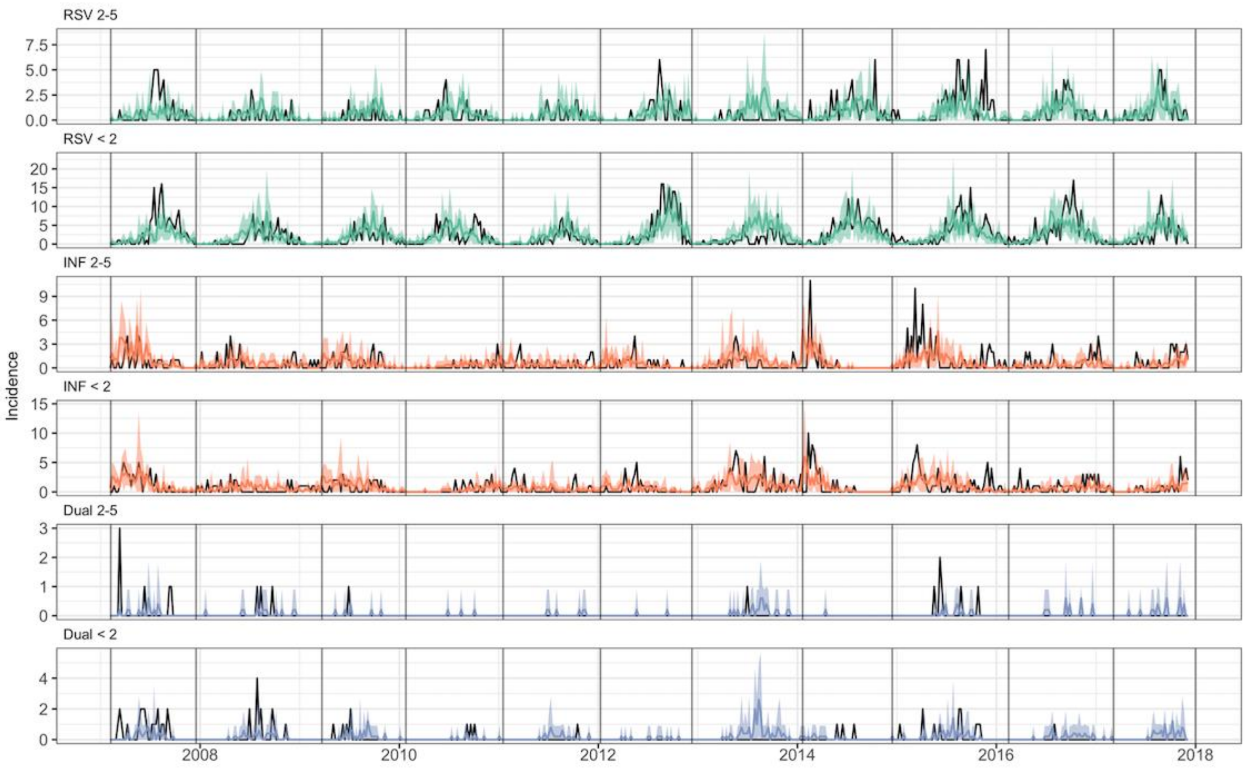

Figure S10: Model Fit at the moderate interaction mode: Black lines are the data, coloured lines are the 95% CrI posterior predictive interval and the mean. Panels show the fit by age group and Virus, and samples were included that had a value for the iinteraction parameter higher than 0.2

(iii) showing profile likelihoods for rho, to clearly show how evidence for the estimates compare.

Thank you for the suggestion of demonstrating the fit using profile likelihoods. We have added this to the supplement as follows:

> *<< Figure S11 shows the profile likelihoods of the interaction parameter, at the two different modes. Samples were taken from the posterior from each mode, and the value of the interaction parameter was then changed to values between 0 and 1, with an interval of 0.02. The log likelihood of each of these points was calculated, and the median and 95% credible interval was then plotted. Infinite values of the log likelihood were excluded from the calculation of the median and credible interval.*

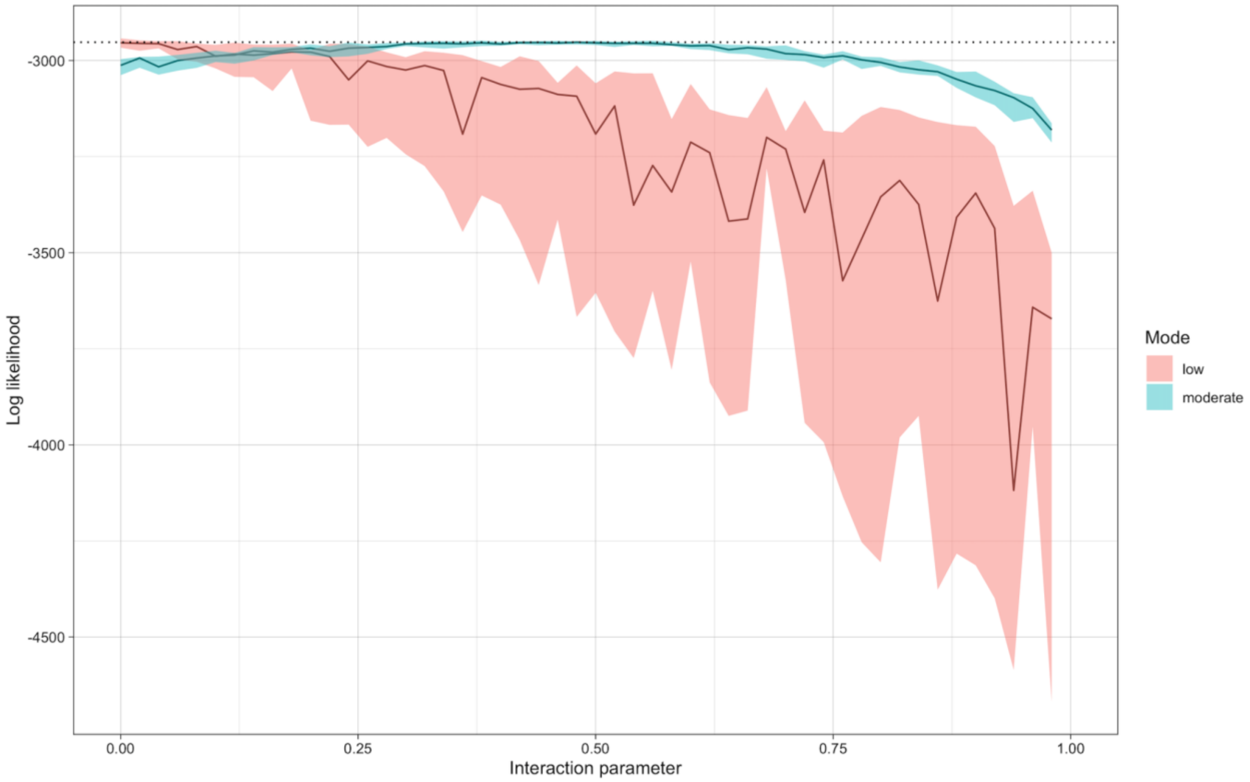

Figure S11: Profile likelihoods of the interaction parameter: Lines indicate the median and ribbons the 95% CrI by mode. The dotted line is placed at the maximum value of the median across both modes. >>

Minor points/comments:

Abstract:

Line 24: Unclear what "heterologous ecological changes" means. Suggest simplifying the language.

This has been changed to: << *Influenza and Respiratory Syncytial Virus (RSV) interact within their host posing the concern for impacts on heterologous viruses following vaccination* >>

Lines 31-33: The statement seems confusing or contradictory. Is this meant to suggest that you data cannot distinguish between

Yes, we are suggesting here that given the available data, we cannot distinguish between them. We have clarified the sentence as follows:

<<The data supported either a 41% (95%CrI: 36 - 54) reduction in susceptibility following infection and for 10.0 days (95%CrI 7.1 -12.8) thereafter, or no change in susceptibility following infection.>>

Lines 33-35: I do not understand what the two respective scenarios with 7.2 and 16.6 fold increases are -- perhaps something is missing?

We thank the reviewer for noticing this bad phrasing. We have updated this sentence to be clearer:

<< We estimate that co-infection increased the probability for an infection in <2y old children to be reported 7.2 fold (95%CrI 5.0 - 11.4); or 16.6 fold (95%CrI 14.5 - 18.4) in the moderate or low interaction scenarios. >>

Background:

One thing to point out in describing the two potential types of interactions, increased severity of co-infections and protective effect of influenza on RSV, is also the timing of those.

Clarifying in these studies the evidence for concurrent infections vs consecutive ones, would be helpful to the readers.

We thank the reviewer for this comment and have added the impact of timings more explicitly.

<< .. The impact of vaccination may be enhanced if concurrent co-infections increases the propensity of severe disease beyond that of either pathogen [...]

Cross-protection following a primary infection could occur through a variety of mechanisms including viral competition for resources in the host(16), the activation of the innate immune system such as through toll-like receptors (TLRs) 3 and 7(17,18) or short term immune memory through surviving cells in an antiviral state (e.g. epithelial cells following influenza infection(19)). These interactions could result in a reduction in subsequent infection with the other viruses, and estimates of the duration of cross-protection and its biological pathway vary. Experimental infection of ferrets estimated less than 2 weeks protection between influenza A and B viruses(20), yet cells forming the respiratory epithelium can survive in a state of heightened antiviral activation for 3 to 12 weeks after influenza A infection, with waning of the conferred protection observable at 6 weeks(19). >>

Perhaps useful to stipulate the specific types of cross-protection you are describing, particularly those in which two viral agents are not related antigenically.

We apologize but it is not clear to us here what the reviewer is suggesting.

Methods

It would useful to know the population denominators for Nha Trang.

This has been added as requested:

> *<< In brief, children younger than 5 years old who resided in 16 out of the 27 communes of Nha Trang (resident population of 210’739) [...] >>*

It is unclear how seasonality is modeled, if it is at all?

Seasonality has not been directly included in the model. Instead, the proportion susceptible at the start of each season has been fit. This has been stated more clearly in the manuscript:

> *<<We model the persisting immunity at the start of each season individually, with an initial proportion infected with each virus. This allows for a different immunity profile at the start of each season, without needing to account for waning of immunity, dynamics across virus subtypes and seasonality specifically.>>*

Figure 1: Age groups do not include 4-5 years?

This is correct, as we are looking at the under 5 population. Therefore our two age groups are ages 0-1 and ages 2-4. We have checked the manuscript for consistency in this regard.

Figure 2: Part of this figure is illegible, especially panel C, model diagram, both due to smal text size and the quality of the figure.

We thank the reviewer for this point and agree with it! The figure has been split up into 3 separate figures, allowing more detail and larger text across the figures.

- Figure 3-2: The Model fit (previously subplot A)
- Figure 3-3: Model diagnostics (previously subplot B and D)
- Figure 3-4: Vaccination impacts (previously subplot E)
-

The model diagram has been moved to the supplement.

Reviewer #3: Overall I think the authors present a strong, well-written, and interesting paper that will help improve our understanding of the interactions between various respiratory pathogens, and the implications for public health. I should make clear that I am not an expert in mathematical modeling, though what the authors describe having done seems reasonable to me. I do have a few minor comments/suggestions for the authors that I think will help improve the overall clarity and readability of the manuscript.

We thank the reviewer for these positive comments!

Comments:

Pg 7 (lines 62-63): This study was only in adults. The evidence in children has been far more mixed. Edit: I see you addressed this later on!

As noted by the reviewer, this has already been addressed in the manuscript.

Pgs 10-11 (lines 144-145) and pg 11 (lines 162-163): Upon first reading these sentences they appear to contradict each other. After looking at it a bit more closely it becomes clear that they do not, but I would recommend revising these sentences to make this more apparent. Your language in the discussion (pg 22, lines: 343-345) was particularly helpful in understanding the reasoning behind your approach, but addressing this earlier in the methods of the paper would be helpful.

Thanks for these comments. We have clarified the sentences mentioned that appear contradictory as follows, as well as adding a sentence to the methods to help with the understanding of the concepts in the paper.

> *<< There is no difference in inherent susceptibility by age for influenza [...]*
>
> *We model the persisting immunity to influenza at the start of each season individually, with an initial proportion susceptible. This allows for a different immunity profile at the start of each season, without needing to account for waning of immunity, dynamics across virus subtypes and seasonality specifically. (25). Due to infections in previous influenza seasons and potential vaccination, susceptibility to influenza is assumed to decline exponentially at rate with age (see supplement section 6). This is modelled as non-leaky cross-protection, due to the combination of different exposures. >>*

Pg 15 (lines: 209-212): The reasoning behind the second sensitivity analysis is a bit confusing as it is currently written. I would suggest the authors revise these sentences a bit to make this clearer.

We thank the reviewer for this suggestion. This has been rephrased:

> *<< In addition, we ran a version of the model which did not allow an increased reporting rate for dual infections, as it has been reported that in this setting there is no increased severity of dual infections among hospitalised children(35). Instead, it was assumed that the reporting rate for dual infections was the same as that for RSV-only infections. >>*

Pg 19 figure 2A: It appears that the dual infections tended to cluster in certain years which might indicate the importance of subtype specific interactions. While I appreciate the substantial complexity that influenza and RSV subtypes would add to the model, if you have data on the influenza and/or RSV subtypes that tended to predominate in years where dual infections were more/less frequently observed this would be quite helpful to include in the descriptive analysis.

We agree that this would be an interesting addition, however we do not have access to this information. However, we have added it to the discussion.

> *<< In addition, the dual infections appear to cluster in certain years, and a different explanation of this could be interaction between different viral subtypes each year. >>*

Pgs 20-21 (lines: 310-313): The setting of this analysis I think is a strength as many of the countries where respiratory viruses circulate at low levels year-round are the same countries with limited influenza vaccine uptake.

We thank the reviewer for this positive comment. We agree this is a strength and have highlighted this in the discussion:

> *<< These location-specific features will need to be considered when generalising the findings. However, in general, the setting of this study is a strength of the paper, as many of the countries where respiratory viruses circulate at low levels year-round are the same countries with limited influenza vaccine uptake. >>*
